# Ultrafast Charge Transfer on Ru‐Cu Atomic Units for Enhanced Photocatalytic H_2_O_2_ Production

**DOI:** 10.1002/adma.202406748

**Published:** 2025-02-18

**Authors:** Chengyang Feng, Jumanah Alharbi, Miao Hu, Shouwei Zuo, Jun Luo, Hassan S. Al Qahtani, Magnus Rueping, Kuo‐Wei Huang, Huabin Zhang

**Affiliations:** ^1^ Center for Renewable Energy and Storage Technologies (CREST) Physical Science and Engineering Division King Abdullah University of Science and Technology (KAUST) Thuwal 23955‐6900 Kingdom of Saudi Arabia; ^2^ KAUST Catalysis Center (KCC) Physical Science and Engineering Division King Abdullah University of Science and Technology (KAUST) Thuwal 23955‐6900 Kingdom of Saudi Arabia; ^3^ State Key Laboratory of Featured Metal Materials and Life‐cycle Safety for Composite Structures MOE Key Laboratory of New Processing Technology for Nonferrous Metals and Materials School of Resources Environment and Materials Guangxi University Nanning 530004 China; ^4^ EXPEC Advanced Research Centre Saudi Aramco Dhahran 31311 Saudi Arabia

**Keywords:** 2D MOFs, bi‐functional unit, H_2_O_2_ photosynthesis, integrated photocatalyst, charge transfer

## Abstract

Photosensitizer‐assisted photocatalytic systems offer a solution to overcome the limitations of inherent light harvesting capabilities in catalysts. However, achieving efficient charge transfer between the dissociative photosensitizer and catalyst poses a significant challenge. Incorporating photosensitive components into reactive centers to establish well‐defined charge transfer channels is expected to effectively address this issue. Herein, the electrostatic‐driven self‐assembly method is utilized to integrate photosensitizers into metal–organic frameworks, constructing atomically Ru‐Cu bi‐functional units to promote efficient local electron migration. Within this newly constructed system, the [Ru(bpy)_2_]^2+^ component and Cu site serve as photosensitive and catalytic active centers for photocarrier generation and H_2_O_2_ production, respectively, and their integration significantly reduces the barriers to charge transfer. Ultrafast spectroscopy and in situ characterization unveil accelerated directional charge transfer over Ru‐Cu units, presenting orders of magnitude improvement over dissociative photosensitizer systems. As a result, a 37.2‐fold enhancement of the H_2_O_2_ generation rate (570.9 µmol g^−1^ h^−1^) over that of dissociative photosensitizer system (15.3 µmol g^−1^ h^−1^) is achieved. This work presents a promising strategy for integrating atomic‐scale photosensitive and catalytic active centers to achieve ultrafast photocarrier transfer and enhanced photocatalytic performance.

## Graphical abstract

[Ru(bpy)_2_]^2+^ photosensitive components are integrated into the Cu‐HHTP MOF, coordinating with the Cu‐O_4_ sites to assemble a bi‐functional unit, thereby facilitating efficient local electron migration. Compared to the dissociative photosensitizer system, this newly constructed configuration exhibits a significant increase in the efficiency of photoinduced charge carrier transfer, resulting in a 37.2‐fold enhancement in the photosynthesis of H_2_O_2_.

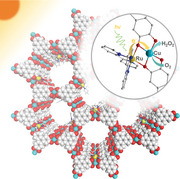

## 1 Introduction

Hydrogen peroxide (H_2_O_2_) stands out as a valuable environmentally friendly oxidant and a promising liquid fuel, generating only water (H_2_O) and oxygen (O_2_) as by‐products, and is regarded as one of the most crucial chemicals in the 21st century.^[^ ^1^, ^2^, ^3^ ^]^ Presently, the anthraquinone method dominates industrial H_2_O_2_ production, constituting over 95% of the process. However, this approach necessitates explosive hydrogen (H_2_) gas and expensive platinum (Pt) catalysts, and also generates toxic organic by‐products, posing a threat to the ecological environment.^[^ ^4^, ^5^, ^6^ ^]^ The artificial photosynthesis production of H_2_O_2_ through the 2e^–^ O_2_ reduction reaction (ORR, O_2_ + 2e^–^ + 2H^+^ → H_2_O_2_) is acknowledged as a green, safe, and energy‐efficient method for H_2_O_2_ production, capturing significant attention over the past decade.^[^ ^7^, ^8^, ^9^, ^10^, ^11^, ^12^ ^]^ Despite considerable efforts, the current activity of photocatalytic H_2_O_2_ synthesis systems falls short of practical requirements.^[^ ^13^ ^]^ Thus, the development of high‐performance photocatalytic systems holds great significance in advancing this field.

The efficiency of artificial photosynthesis systems is chiefly determined by factors including the light‐harvesting capability of the photosensitive unit, the reactivity of the catalytic unit, and the charge transfer efficiency between the photosensitive and the catalytic unit.^[^ ^14^, ^15^, ^16^ ^]^ In traditional photocatalytic systems, the photosensitizer is dispersed dissociative,^[^ ^17^, ^18^ ^]^ hindering direct contact with the photocatalyst and thus severely impeding charge transfer. Hence, to achieve comparable catalytic activity, it typically requires adding several times the amount of photosensitizer compared to the catalyst to compensate for the low charge transfer efficiency,^[^ ^19^, ^20^, ^21^, ^22^ ^]^ thereby constraining the practical application. Integrating photosensitizers and catalysts into tightly connected dual active units is anticipated to offer an ideal solution (Figure S1, Supporting Information). The interconnected photosensitive and catalytic units facilitate efficient electron transfer, expediting electron extraction and minimizing energy loss. However, constructing such structures is challenging because most photocatalytic materials lack sites to anchor photosensitizers. Metal–organic frameworks (MOFs) leverage their open configuration space and flexible coordination environment, offering a tunable platform for the precise design of integrated photosensitizers and reaction units.^[^ ^23^, ^24^, ^25^, ^26^, ^27^, ^28^, ^29^, ^30^ ^]^ There are numerous successful cases of integrating photosensitizer components into MOFs to achieve photocatalytic reactions.^[^ ^31^ ^]^ However, outcomes where the photosensitizer sites are directly assembled with active reaction sites to maximize the efficiency of photogenerated charge carrier transfer are quite rare. Two‐dimensional π‐conjugated MOFs, typified by Cu‐HHTP (HHTP = 2,3,6,7,10,11‐hexahydroxytriphenylene), feature a through‐channel structure facilitating the internal assembly of photosensitizers,^[^ ^32^, ^33^, ^34^, ^35^, ^36^, ^37^ ^]^ while their outstanding conductivity and accessible Cu metal sites enhance the acceleration of photogenerated electron transfer.^[^ ^38^, ^39^, ^40^, ^41^ ^]^ By manipulating the photosensitizer integration on the Cu site of Cu‐HHTP, it is anticipated that a well‐defined photosensitive‐reactive dual‐active unit can be achieved, thereby enabling efficient excitation and extraction of photogenerated charges.

Herein, we demonstrate that [Ru(bpy)_2_]^2+^ (abbreviated as Rubpy, bpy = 2,2′‐bipyridine) can be assembled into the channels of Cu‐HHTP through a facile one‐step coordination chemistry method to construct a photosensitive‐reactive bi‐functional unit to achieve ultra‐fast local photogenerated electron transfer. The Ru‐Cu coordination structure is designed to enable efficient production and transfer of photoinduced charges through the optical (Rubpy component) and catalytic (Cu site) centers. We elucidate the coordination configurations of Ru‐Cu units and reveal the interactions between them. As a result, the optimized Ru@Cu‐HHTP photocatalyst exhibits a 37.2‐fold increase in the photocatalytic H_2_O_2_ production rate compared to the original Cu‐HHTP under the assistance of the same amount of [Ru(bpy)_3_]^2+^ photosensitizer (abbreviated as **Ru**). Experimental characterization and theoretical calculations have confirmed that the created Ru‐Cu local structure can function as a swift extraction channel for photogenerated electrons, facilitating the transfer of photogenerated electrons from the photosensitive center to the Cu site. This study presents a scalable strategy for integrating photosensitizers and catalysts to construct photosensitive‐catalytic bi‐functional active centers, enabling efficient photocatalytic H_2_O_2_ synthesis.

## 2 Results and Discussion

**Figure 1:**
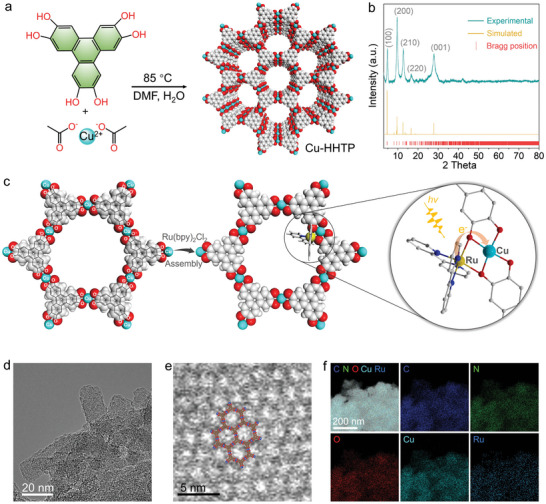
a) Illustration of synthetic routes of two‐dimensional MOF Cu‐HHTP. b) Experimental and simulated PXRD images of Ru@Cu‐HHTP. c) Self‐assembly of [Ru(bpy)_2_]^2+^ into Cu‐HHTP to create the photocatalyst with bi‐functional unit. d) TEM image of Ru@Cu‐HHTP. e) High‐resolution TEM image of the 2D honeycomb structures. f) Elemental mappings of Ru@Cu‐HHTP.

The Cu‐HHTP is synthesized via a facile hydrothermal method, where the organic ligand HHTP react with Cu^2+^ in a mixed solvent containing water and dimethylformamide at 85 °C (**Figure** **1**a). [Ru(bpy)_2_]^2+^ is incorporated into Cu‐HHTP through a facile coordination self‐assembly and is adjacent to the Cu‐O_4_ site (Figure 1c), thus promoting the transfer of photogenerated charges. Powder X‐ray diffraction (PXRD) patterns reveal that the crystalline structure of the Cu‐HHTP MOF remains unchanged throughout the self‐assembly process (Figure S2, Supporting Information). The X‐ray photoelectron spectroscopy (XPS) results confirm the robust coordination of oxygen atoms in Cu‐HHTP with Ru ions, as evidenced by the energetic upshift of the O 1s peaks (Figures S3–S5, Supporting Information).^[^ ^42^ ^]^ Moreover, the presence of the Ru 3p signal is distinctly observed in Ru@Cu‐HHTP, affirming the successful assembly of the Ru photosensitive unit (Figure S3d, Supporting Information). Furthermore, in comparison to the host Cu‐HHTP MOF, the UV‐vis absorption spectrum of Ru@Cu‐HHTP demonstrates an obvious red‐shift, indicating that the introduction of photosensitive units improves the overall light harvesting (Figure S6, Supporting Information).

Transmission electron microscopy (TEM) unveils that the samples consist of nanocrystal with average dimensions of 27 nm in length and 9 nm in width (Figure 1d; Figure S7, Supporting Information). The small size and uniform size distribution of the nanocrystals contribute to enhanced activity and better control of the reaction process.^[^ ^43^ ^]^ A honeycomb structure with a pore size of ≈1.9 nm has been observed by high‐resolution TEM (Figure 1e), corresponding to face‐centered hexagonal packing of (100) and (200) peaks in the PXRD pattern (Figure 1b). It is noteworthy that the introduction of the photosensitive unit only causes a slight reduction in the specific surface area and porosity of Cu‐HHTP, indicating that its high specific surface area and open pore structure are well preserved, ensuring effective entry of O_2_ into the reaction site (Figure S8, Supporting Information). The elemental composition of Ru@Cu‐HHTP is further verified by energy dispersive X‐ray spectroscopy (EDS, Figure S9, Supporting Information) and elemental mapping (Figure 1f). The uniform distribution of Ru elements on Cu‐HHTP is confirmed, demonstrating the successful assembly of Ru photosensitive components. Additionally, ICP‐MS analysis corroborate the Cu/Ru ratio in the samples, as presented in Table S1 (Supporting Information), the maximum Ru assembly ratio in the Cu‐HHTP MOF is Cu:Ru = 1:0.16.

**Figure 2:**
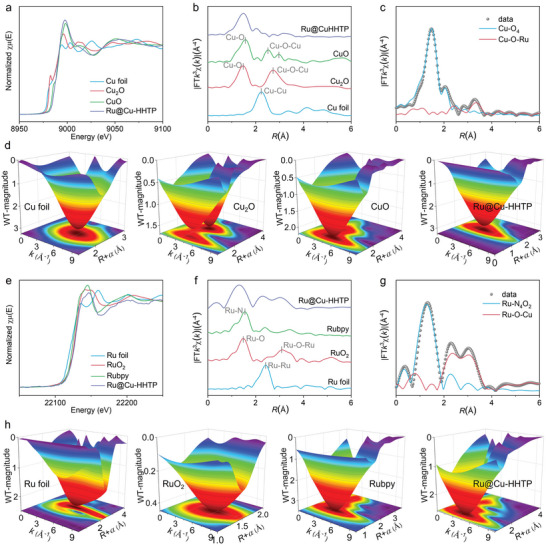
a) Cu K‐edge XANES experimental spectra and b) Cu K‐edge Fourier‐transformed EXAFS spectra of Ru@Cu‐HHTP, CuO, Cu_2_O, and Cu foil. c) EXAFS fitting at Cu K‐edge of the Ru@Cu‐HHTP in R space based on the model obtained from DFT simulation. d) Wavelet transform of Cu K‐edge EXAFS spectra for Cu foil, Cu_2_O, CuO, and Ru@Cu‐HHTP. e) Ru K‐edge XANES experimental spectra and (f) Ru K‐edge Fourier‐transformed EXAFS spectra of Ru@Cu‐HHTP, Rubpy, RuO_2,_ and Ru foil. g) EXAFS fitting at Ru K‐edge of the Ru@Cu‐HHTP in R space based on the model obtained from DFT simulation. h) Wavelet transform of Ru K‐edge EXAFS spectra for Cu foil, Cu_2_O, CuO and Ru@Cu‐HHTP.

The atomic structure and coordination environment for Ru‐Cu bi‐functional units in Ru@Cu‐HHTP have been further characterized by X‐ray absorption fine structure (XAFS) spectroscopy. **Figure** **2**a illustrates the Cu K‐edge X‐ray absorption near edge structure (XANES) spectra of Ru@Cu‐HHTP and corresponding reference samples, including Cu foil, Cu_2_O, and CuO. The linear fitting results indicate the valence state of the Cu species in Ru@Cu‐HHTP is +1.7 (Figure S10a, Supporting Information), proving the main presence of Cu^2+^ and a partial Cu^1+^. The Fourier‐transformed extended X‐ray absorption fine structure (EXAFS) spectrum of Ru@Cu‐HHTP exhibits a main peak at 1.48 Å (Figure 2b), which can be well attributed to the contributions of primary‐coordinated Cu‐O bonds. The wavelet transform (WT) spectra confirm the absence of a secondary correlation from the scattering of cluster or oxide (Figure 2d). Furthermore, the EXAFS fitting results revealed the local fine configurations of the Cu_1_‐O_4_ coordination in the first coordination shell of Cu species and the Cu_1_‐O‐Ru_0.2_ coordination in the second coordination shell (Figure 2c), indicating that approximately one‐fifth of the Cu sites are coordinated with Ru, consistent with the results obtained from ICP analysis. In addition, the Ru K‐edge XANES spectra of Ru@Cu‐HHTP is also conducted, the resemblance of the white‐line peak between Ru@Cu‐HHTP and Rubpy indicates a similar coordination structure of Ru atoms (Figure 2e). The fitting results suggest that the oxidation state of Ru species in Ru@Cu‐HHTP is ≈+2.1 (Figure S10b, Supporting Information), which is consistent with the oxidation state of the precursor Rubpy. Comparison with Ru foil, Rubpy, and RuO_2_ references, the EXAFS spectra reveal Ru species with Ru‐nonmetal coordination in the first shell and a Ru‐O‐metal coordination configuration resembling RuO_2_ in the second shell (Figure 2f). To bolster these findings, WT contour plots of Ru foil, RuO_2_, Rubpy, and Ru@Cu‐HHTP have been scrutinized (Figure 2h). It is evident that Ru@Cu‐HHTP exhibits WT plots highly consistent with Rubpy, indicating a similar Ru coordination structure therein. Further EXAFS fitting results confirm the coordination structure of Ru species, consisting of Ru_1_‐N_4_O_2_ first coordination and Ru‐O‐Cu in second shell (Figure 2g). The Ru‐N_4_ coordination is retained from the original bpy structure, while the Ru‐O_2_ coordination arises from the assembly with Cu‐HHTP. Additionally, a Cu‐HHTP MOF model containing Ru‐Cu units is constructed. The locally optimized structure obtained through density functional theory (DFT) calculation is illustrated in Figure S11 (Supporting Information), with the obtained coordination and bond length information matching the XAFS analysis results. At this stage, the above characterizations and analyzes have verified the successful construction of Ru‐Cu bi‐functional units in Ru@Cu‐HHTP.

**Figure 3:**
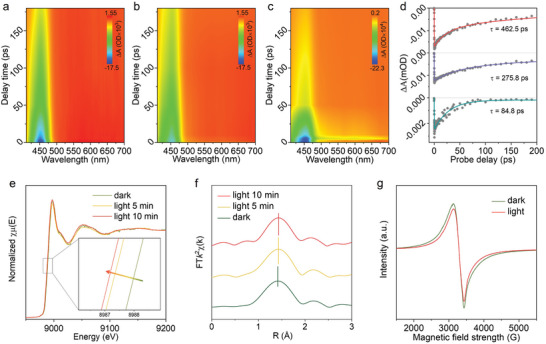
Transient absorption contour plot of a) **Ru**, b) [**Ru** + Cu‐HHTP], and c) Ru@Cu‐HHTP. d) Transient absorption decay kinetics of **Ru**, [**Ru** + Cu‐HHTP] and Ru@Cu‐HHTP. e) In situ XANES Cu k‐edge spectra of Ru@Cu‐HHTP under dark and light irradiation conditions. f) Changes in R space of Ru@Cu‐HHTP under dark and light irradiation conditions. g) In situ EPR spectra of Ru@Cu‐HHTP under dark and light irradiation conditions.

To reveal the charge transfer on Ru‐Cu bi‐functional units, femtosecond time‐resolved transient absorption (fs‐TA) spectroscopy has been conducted to investigate the ultra‐fast excited‐state decay dynamics. As illustrated in **Figure** **3**a, the intuitive pseudo‐color fs‐TA spectra of the photosensitizer **Ru** exhibit a distinct negative signal indicative of ground state bleaching ≈450 nm, indicating the significant recombination of photogenerated charges (Figure S12a, Supporting Information).^[^ ^44^ ^]^ The [**Ru** + Cu‐HHTP] system exhibits a ground‐state bleaching peak with the same profile as that of the individual **Ru** photosensitizer (Figure 3b), indicating that the photoexcitation process in the [**Ru** + Cu‐HHTP] system is still primarily dominated by the excitation and relaxation of photogenerated electrons on the **Ru** photosensitizer. Although the ground‐state bleaching peak of [**Ru** + Cu‐HHTP] shows a certain degree of reduction compared to **Ru**, which can be attributed to the transfer of photogenerated electrons from the photosensitizer **Ru** to Cu‐HHTP,^[^ ^18^ ^]^ the primary pathway for photogenerated charge recombination remains unchanged (Figure S12b, Supporting Information). This results in low utilization efficiency of photogenerated charge carriers in the [**Ru** + Cu‐HHTP] system. In comparison, two significant spectral changes were observed on Ru@Cu‐HHTP (Figure 3c). First, the decay of the ground‐state bleaching peak ≈450 nm accelerated notably, indicating rapid extraction of photogenerated electrons. Second, a rapidly decaying excited‐state absorption signal appeared in the 500 to 700 nm range, attributed to a secondary transition of the excited‐state electrons, which was absent in both the individual **Ru** photosensitizer and the [**Ru** + Cu‐HHTP] mixed system. These results indicate that the pathway for photogenerated charge excitation and transfer in the Ru‐Cu unit has fundamentally shifted compared to the **Ru** and [**Ru** + Cu‐HHTP] systems (Figure S12c, Supporting Information). Therefore, it is reasonable to conclude that the photogenerated electrons excited on the photosensitizer component can efficiently transfer to the Cu sites via two pathways: (i) rapid extraction of photogenerated electrons through the Ru‐O‐Cu pathway, connected via metal‐oxygen bonds, and (ii) a secondary transition of excited‐state electrons to the Cu sites via excited‐state absorption.^[^ ^45^, ^46^ ^]^ Furthermore, the fs‐TA kinetic plots at 450 nm for the three aforementioned test results have been subjected to further fitting using a biexponential equation (Figure 3d). The results underscore the ultrafast efficiency of electron transfer over the Ru‐Cu units on Ru@Cu‐HHTP catalysts, representing an order of magnitude improvement over the dissociative photosensitizer. Subsequently, time‐resolved photoluminescence spectra were also conducted to verify the ultrafast photogenerated charge transfer in Ru@Cu‐HHTP. As shown in Figure S13 (Supporting Information), the [**Ru** + Cu‐HHTP] system shows only limited enhancement in the fluorescence decay rate of **Ru**. However, when **Ru** is assembled onto Cu‐HHTP to form the Ru‐Cu unit, the fluorescence decay is significantly accelerated. Given that faster decay represents higher efficiency, this confirms that the Ru‐Cu unit enables ultrafast extraction of photogenerated charge carriers.

Then, the XAFS spectra are employed for a more detailed investigation of the photogenerated electron transfer process within the Ru@Cu‐HHTP system. As depicted in Figure 3e, with prolonged illumination, the Cu K‐edge shifts toward lower energy (shifted by 0.9 eV), suggesting a gradual reduction in the valence state of Cu. This observation implies the transfer of photogenerated electrons from the photosensitive unit to the Cu site. Moreover, the corresponding k‐space data reveals that as photogenerated electrons transfer to Cu, there is an expansion in the Cu─O coordination, which proves advantageous for the adsorption and reduction of O_2_ molecules (Figure 3f).^[^ ^47^ ^]^ The EPR test conducted under illumination provided additional evidence supporting the transfer of photogenerated electrons to Cu sites. As displayed in Figure 3g, the decreased EPR signal confirmed that light irradiation caused the conversion of a portion of EPR‐active Cu^2+^ into the EPR‐silent species, presumably Cu^+^,^[^ ^48^, ^49^, ^50^ ^]^ where electrons have accumulated and constitute the active site responsible for driving the reduction of O_2_.

**Figure 4:**
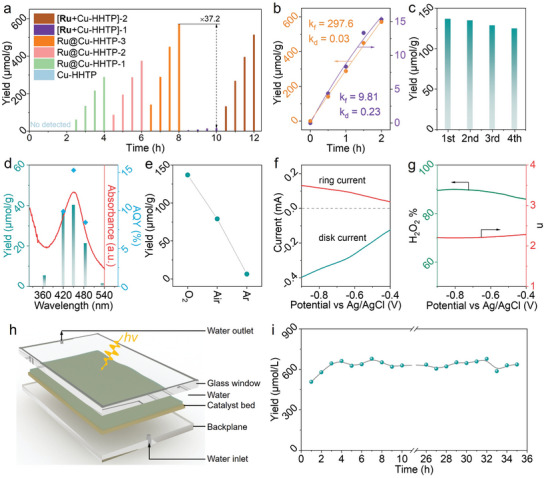
a) Photocatalytic H_2_O_2_ production over pristine Cu‐HHTP, Ru@Cu‐HHTP samples, and Cu‐HHTP with **Ru** photosensitizer. b) The fitted formation rate constants (*K_f_*) and decomposition rate constants (*K_d_*) of Ru@Cu‐HHTP and Cu‐HHTP with **Ru** photosensitizer. c) Cycling stability of the Ru@Cu‐HHTP catalysts. (d) Wavelength‐dependent H_2_O_2_ generation rates and AQY of Ru@Cu‐HHTP. e) Photocatalytic H_2_O_2_ production over Ru@Cu‐HHTP under different conditions. (f) RRDE voltammograms of Ru@Cu‐HHTP in O_2_‐saturated solution. g) Percentage of peroxide and the electron transfer numbers of Ru@Cu‐HHTP at various potentials. h) Structural diagram of photocatalytic flow cell. i) Long timescale H_2_O_2_ production over the Ru@Cu‐HHTP catalyst in flow cell.

The photocatalytic performances for H_2_O_2_ production of Ru@Cu‐HHTP have been evaluated in an O_2_‐equilibrated condition under visible light irradiation, along with the catalytic activity of the [**Ru** + Cu‐HHTP] system (**Figure** **4**a; Figure S14, Supporting Information). Ru@Cu‐HHTP exhibits outstanding photocatalytic ORR activity for H_2_O_2_ production, and this activity increases with higher Ru loading, affirming that the Ru‐Cu bi‐functional unit enhances the catalytic process. The maximized H_2_O_2_ production rate of 570.9 µmol g^−1^ h^−1^ is achieved on Ru@Cu‐HHTP, which is 37.2 times of the [**Ru** + Cu‐HHTP] system using the same amount of photosensitizer (calculated based on Ru element). To achieve a yield comparable to that of Ru@Cu‐HHTP in the [**Ru** + Cu‐HHTP] system, ≈15 times more photosensitizer needs to be added. The notable disparity in catalytic activity between the two systems stems from their distinct mechanisms of photogenerated electron transfer. In the [**Ru** + Cu‐HHTP] system, photosensitizer **Ru** donates photogenerated electrons to Cu‐HHTP via random collisions, which is greatly constrained. However, in Ru@Cu‐HHTP, photogenerated electrons are directly transferred between adjacent coordinated Ru‐Cu units, markedly enhancing efficiency (Figure S15, Supporting Information). Considering that the accumulation of H_2_O_2_ is governed by the competitive kinetics of its formation (K_f_) and decomposition (K_d_), we then derive the values of K_f_ and K_d_ constants by assuming zero‐order kinetics for H_2_O_2_ formation and first‐order kinetics for H_2_O_2_ decomposition. As displayed in Figure 4b, Ru@Cu‐HHTP exhibit 30.4 times higher H_2_O_2_ generation rate with 1/8 the H_2_O_2_ decomposition rate compared to [**Ru** + Cu‐HHTP] system, the assembly of photosensitizers can not only enhance the catalytic activity but also inhibit the re‐decomposition of H_2_O_2_. Additionally, control experiments confirmed that dissociative **Ru** species induce the decomposition of H_2_O_2_, whereas the assembly and fixation of **Ru** inhibit this process (Figure S16, Supporting Information). The assembled Ru@Cu‐HHTP catalyst also has excellent stability, showing no significant loss of catalytic activity after four consecutive photocatalytic runs (Figure 4c).

Then the reaction is carried out with monochromatic light of different wavelengths, the H_2_O_2_ formation rate qualitatively follows the characteristic absorption spectrum of Rubpy photosensitizer, confirming that the reaction is indeed triggered by the light excitation of Ru‐Cu bi‐functional units (Figure 4d). The calculated apparent quantum yield (AQY) values at 420, 450, and 485 nm are 9.8%, 15.3%, and 8.4%, respectively. The effects of incident light intensity and catalyst dosage on the AQY were further evaluated. Undoubtedly, higher incident light intensity enhances the net production rate of H_2_O_2_. However, it also leads to an increased proportion of photon dissipation, resulting in a decline in AQY (Figure S17, Supporting Information). On the other hand, increasing the catalyst dosage can improve AQY, but the shielding effect caused by particle aggregation reduces the activity per unit of catalyst (Figure S18, Supporting Information). Additionally, the solar‐to‐chemical conversion efficiency (SCC) under simulated sunlight is 0.13%, further confirming the excellent photocatalytic performance of Ru@Cu‐HHTP. The production of H_2_O_2_ is significantly inhibited under air or argon atmosphere, proving that dissolved oxygen is a necessary raw material for H_2_O_2_ production (Figure 4e). To further understand the oxygen reduction mechanism, rotating ring‐disk electrode (RRDE) tests have been conducted to investigate the number of transferred electrons (n) and the selectivity toward H_2_O_2_ during the oxygen reduction process (Figure 4f). The fitting results confirm that the oxygen reduction process on Ru@Cu‐HHTP via a 2e^−^ transfer mechanism, with up to 90% selectivity toward H_2_O_2_ (Figure 4g). Based on the high activity and stability of the constructed Ru@Cu‐HHTP catalyst, long‐term operation of Ru@Cu‐HHTP has been evaluated in a continuous flow reactor (Figure 4h). It can be observed that stable H_2_O_2_ production is maintained over a continuous 35 h operation (Figure 4i), with no apparent detachment of the assembled Ru photosensitive components from the catalyst after reaction (Figures S19–S21 and Table S1, Supporting Information).

**Figure 5:**
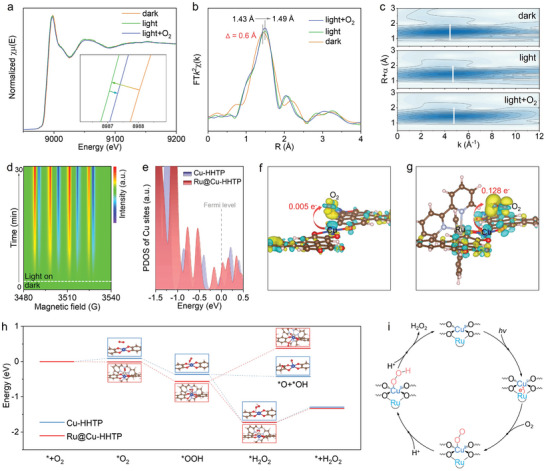
a) In situ XANES Cu k‐edge spectra of Ru@Cu‐HHTP photocatalyst treated successively with dark, light, and light + O_2_ condition b) and the corresponding R space spectra. c) Wavelet transform of Cu K‐edge EXAFS spectra for Ru@Cu‐HHTP under different conditions. d) In situ EPR signals of DMPO‐•OOH over Ru@Cu‐HHTP under light irradiation. e) Calculated PDOS of Cu‐HHTP and Ru@Cu‐HHTP. Calculated charge density distribution of O_2_ molecules absorbed on Cu site of f) Cu‐HHTP and g) Ru@Cu‐HHTP. h) Calculated energy profiles of O_2_ reduction to H_2_O_2_ over Cu‐HHTP and Ru@Cu‐HHTP. i) Proposed mechanism of photocatalytic H_2_O_2_ production over Ru‐Cu dual active units on the Ru@Cu‐HHTP photocatalyst.

To gain insight into the activation of O_2_ on Ru‐Cu units, in situ XAFS measurements are performed. As depicted in **Figure** **5**a, illumination induces a leftward shift in the absorption edge, consistent with previous discussions, attributable to the transfer of photo‐generated electrons from the photosensitive components to the Cu sites. However, upon the introduction of O_2_ into the reaction system, the absorption peak shifts back to the right, indicating an increase in the oxidation state of Cu, possibly due to the transfer of photo‐generated electrons from the Cu sites to the adsorbed O_2_. In addition, due to the accumulation of photogenerated electrons on Cu sites, an expansion of the Cu‐O bond in the first coordination layer is observed after illumination (Figure 5b). Upon the introduction of O_2_, the peak intensity increases, indicating a rise in the coordination number of Cu sites. The corresponding structural evolution is more prominently displayed in the WT contour plots (Figure 5c), confirming the adsorption of O_2_ on Cu sites. The above analysis indicates that O_2_ adsorbs on Cu sites and accepts photo‐generated electrons from the photosensitive units for activation. However, capturing the O_2_ reduction intermediate on Cu sites by in situ XAFS is challenging, therefore, in situ EPR capture experiments are conducted. As shown in Figure 5d, there is no signal under dark conditions, while typical •OOH characteristic peaks appear and gradually increase with illumination, indicating the occurrence of a two‐step 2e^−^ ORR reaction (O_2_ + e^−^ + H^+^ → •OOH; •OOH + e^−^ + H^+^ → H_2_O_2_). To further reveal the reaction process accurately and intuitively, in situ diffuse reflectance infrared Fourier transform spectroscopy (DRIFTS) measurements were conducted to identify the real‐time intermediates during photocatalytic H_2_O_2_ production. As displayed in Figure S22 (Supporting Information), the characteristic peak at 925 cm^−1^ corresponds to the O‐O bond of adsorbed oxygen,^[^ ^51^ ^]^ confirming the effective adsorption of O_2_ on Ru@Cu‐HHTP. With extended light exposure, the signals of •O_2_^−^ (1102 cm^−1^) and •OOH (1225 cm^−1^) gradually intensified,^[^ ^52^, ^53^ ^]^ alongside the emergence of the characteristic peak for adsorbed H_2_O_2_ (1365 cm^−1^),^[^ ^54^ ^]^ confirming that O₂ is reduced via a two‐electron pathway, forming ·OOH, which is further converted to H_2_O_2_.

Furthermore, DFT calculations elucidate the activation of O_2_ and the generation of H_2_O_2_ on Ru‐Cu bi‐functional units. First, the density of states (DOS) of Cu species before and after assembling Ru units is compared. Due to the interaction between Ru units and Cu sites, the electron density of Cu species near the Fermi level significantly increases (Figure 5e), indicating the enhancement of reactivity. Moreover, Figure 5f,g depicts the charge density difference of O_2_ adsorption on pristine Cu sites and Ru‐Cu dual units. Compared to Cu‐HHTP, the charge redistribution of O_2_ on Ru‐Cu units is more pronounced, suggesting that the assembly of photosensitive components not only facilitates charge transfer efficiency but also enhances the reactivity of Cu sites. Based on the above results, the free energy changes of the photocatalytic 2e^−^ ORR on the Cu‐HHTP and Ru@Cu‐HHTP are further calculated. As depicted in Figure 5h, the introduction of Ru units facilitates the adsorption of O_2_, reduce the adsorption energy from 0.09 to −0.01 eV. The enhanced O_2_ adsorption capability of Ru‐Cu units further promotes the formation of *OOH, thereby significantly facilitating the conversion of *OOH to H_2_O_2_. More importantly, the assembly of Ru units significantly suppress the thermodynamically favorable *OOH decomposition side reaction (*OOH → *O + *OH), shifting its free energy change from +0.06 to −0.94 eV. Based on the above results, a mechanism for the photocatalytic synthesis of H_2_O_2_ via two‐electron ORR on Ru‐Cu bi‐functional units is proposed (Figure 5i). Under light irradiation, facilitated by the unique dual‐unit configuration, photogenerated electrons swiftly transfer from the Ru photosensitive component to the adjacent Cu reaction site. The activated Cu sites facilitate the adsorption and reduction of O_2_, driving a two‐step 2e^−^ ORR reaction to produce H_2_O_2_. Simultaneously, the decomposition of the crucial intermediate *OOH is inhibited, further promoting H_2_O_2_ generation.

## 3 Conclusion

In summary, we assembled photosensitive components in the 2D Cu‐HHTP, constructing a MOF‐supported photocatalyst with Ru‐Cu bi‐functional active centers to accelerate charge transfer. The Ru units serve as light‐harvesting centers, while the Cu sites provide high reactivity for oxygen reduction to produce H_2_O_2_. In situ characterization and ultrafast spectroscopy revealed the dynamic behavior of photogenerated carriers on Ru‐Cu units. The swift electron transfer from Ru to Cu sites significantly enhances charge separation, thereby leading to excellent catalytic activity at the electron‐rich Cu sites, which greatly facilitates the transformation of O_2_ to H_2_O_2_. Moreover, in addition to the enhanced charge transfer and catalytic activity, the assembly strategy of the photosensitizer effectively suppresses the re‐decomposition of intermediate *OOH and H_2_O_2_. As a result, the integrated photocatalyst achieved a remarkable 37.2‐fold increase in H_2_O_2_ production rate compared to the photocatalytic system using dissociative photosensitizer. Therefore, we believe that the precise construction strategy of photocatalytic units reported in this work provideAuthor: Please check funding information and confirm its correctness.s a promising approach for achieving efficient H_2_O_2_ synthesis, and also offers guidance for future exploration of photocatalysts that integrate photosensitizer, oxidation, and reduction components to accomplish overall reactions.

## Conflict of Interest

The authors declare no conflict of interest.

## Supporting information



Supporting Information

## Data Availability

The data that support the findings of this study are available from the corresponding author upon reasonable request.
