# Supplementary material for: Ultrafast Charge Transfer on Ru‐Cu Atomic Units for Enhanced Photocatalytic H2O2 Production
Source: Adv Mater. 2025 Feb 18;37(12):2406748. doi: 10.1002/adma.202406748 (PMC11937988; doi:10.1002/adma.202406748)
Supplement: Supplementary file 1 — Supporting Information [file ADMA-37-2406748-s001.docx]

**Ultrafast Charge Transfer on Ru-Cu Atomic Units for Enhanced Photocatalytic H_2_O_2_ Production**

*Chengyang Feng, Jumanah Alharbi, Miao Hu, Shouwei Zuo, Jun Luo,** *Hassan S. Al Qahtani, Magnus Rueping, Kuo-Wei Huang, Huabin Zhang**

Dr. C. Feng, J. Alharbi, M. Hu, Dr. S. Zuo, Prof. K.W. Huang, Prof. H. Zhang

Center for Renewable Energy and Storage Technologies (CREST), Physical Science and Engineering Division, King Abdullah University of Science and Technology (KAUST), Thuwal, 23955-6900, Kingdom of Saudi Arabia.

E-mail: huabin.zhang@kaust.edu.sa

Dr. C. Feng, J. Alharbi, M. Hu, Dr. S. Zuo, Prof. M. Rueping, Prof. K.W. Huang, Prof. H. Zhang

KAUST Catalysis Center (KCC), Physical Science and Engineering Division, King Abdullah University of Science and Technology (KAUST), Thuwal 23955-6900, Kingdom of Saudi Arabia.

Prof. J. Luo

State Key Laboratory of Featured Metal Materials and Life-cycle Safety for Composite Structures, MOE Key Laboratory of New Processing Technology for Nonferrous Metals and Materials

School of Resources, Environment and Materials, Guangxi University, Nanning 530004, China

E-mail: junluo@gxu.edu.cn

Dr. H. S. Al Qahtani

EXPEC Advanced Research Centre, Saudi Aramco, Dhahran 31311, Saudi Arabia

**Experimental section**

**Chemicals.** All reagents were commercially available and used without further purification.

**Synthesis of Cu-HHTP.** Mix 2,3,6,7,10,11-hexahydroxytriphenylene (HHTP, 8 mg), Cu(CH_3_COO)_2_·H_2_O (22 mg), N,N-dimethylformamide (DMF, 5 mL) and deionized water (5 mL) uniformly and seal them in a 20 mL vial. Following 30 minutes of sonication, the vials underwent heating at 85 °C for 12 hours. Upon cooling to room temperature, the resulting dark blue powder was gathered through filtration, sequentially washed with acetone, ethanol, and deionized water, and subsequently vacuum-dried at 65°C overnight for further experiments and characterization.

**Synthesis of Ru@Cu-HHTP.** Ru@Cu-HHTP was synthesized through the adsorption and chelation of cis-Dichlorobis(bipyridine)ruthenium(II) and Cu-HHTP. Typically, the Cu-HHTP MOF (100 mg) was dispersed in acetonitrile (20 mL) and stirred for 30 min to prepare the precursor solution. Afterward, cis-Dichlorobis(bipyridine)ruthenium(II) (5 mg, 10 mg, 20 mg, 30 mg) was added to the aforementioned solution and stirred for 24 h at 25 °C under an N_2_ atmosphere. Ru@Cu-HHTP precipitates were collected through centrifugation. The collected solid was washed three times with DMF, ethanol, and DI water, respectively, to ensure the complete removal of uncoordinated cis-Dichlorobis(bipyridine)ruthenium(II), and then dried for 24 hours at 60 °C in a vacuum oven. The Ru loading was regulated by adjusting the dosage of cis-Dichlorobis(bipyridine)ruthenium(II), and the actual Ru loading was verified through inductively coupled plasma-atomic emission spectrometry (Table S1). Once the Ru loading reaches 3.6 wt%, the coordination becomes saturated, and further increasing the dosage of cis-Dichlorobis(bipyridine)ruthenium(II) will not markedly enhance the Ru content in the final catalyst.

**Characterizations.** Powder XRD is characterized by a powder X-ray diffraction instrument (Bruker D8 Advanced A25 diffractometer) with a Cu Kα target (λ = 1.54056 Å) at 40 kV and 40 mA. N_2_ adsorption isotherms were operated using a Micromeritics ASAP 2420 at 77 K. The optical absorption properties of the samples are determined using the diffuse reflection method on a UV-visible light near-infrared spectrometer (Lambda 950). TEM images are obtained on a Titan ST microscope from Thermo Fisher Scientific. The X-ray absorption fine structure (XAFS) spectra (Mn K-edge) are collected at the beamline 1W1B of the Beijing Synchrotron Radiation Facility (BSRF, Beijing) in a fluorescence mode at room temperature. XPS measurements are performed on an ESCALAB 250Xi spectrometer (Thermo Fisher) with a monochromatic Al Kα X-ray source. EPR measurements are obtained at room temperature using a Bruker EMX-10/12 EPR spectrometer operated in the X-band frequency. The inductively coupled plasma atomic emission spectroscopy (ICP-AES) measurements were performed on a PerkinElmer Optima 3300DV (ICP) spectrometer.

**Femtosecond transient absorption measurement.** The femtosecond transient absorption (fs-TA) measurements are conducted using a commercial fs-TA system. A fundamental 800 nm pulse generated by a Coherent Astrella regenerative amplifier served as the pump source. This pulse is used to pump an optical parametric amplifier (Coherent, OperA Solo), producing a frequency-tunable pump beam spanning the visible light region. The pump beam, with a wavelength of 350 nm, is then focused onto the sample. A white-light continuum probe beam is generated by focusing a weaker portion of the fundamental 800 nm beam onto a sapphire window. The sample is positioned at the overlap of the pump beam and the white-light continuum probe beam. All measurements are conducted with samples in water solution using 1 mm quartz cuvettes.

**Photocatalytic measurements.** Typically, 10 mg of as-synthesized photocatalyst was dispersed in 50 mL ethanol aqueous solution (10%). The suspension solutions were stirred for 30 min in the dark with continual O_2_ bubbling after ultrasound treatment for 10 min to reach the absorption–desorption equilibrium. Then the solutions were exposed to visible light provided by a 300 W Xe lamp with a 400 nm cutoff filter. The light source was located at a distance of 10 cm from the reactor, and a continuous magnetic stirrer and cooling water were applied during the experiment. During illumination, 1 mL solution was sampled every quarter hour and filtrated with a 0.45 µm to remove the photocatalyst. Additionally, control groups using free photosensitizer were implemented. The dosage of [Ru(bpy)_3_]Cl_2_ for [**Ru**+Cu-HHTP-1] and [**Ru**+Cu-HHTP-2] were 1.36 mg and 20 mg, respectively.

The amount of H_2_O_2_ was analyzed by iodometry. Typically, 1 mL of 0.1 mol L^−1^ C_8_H_5_KO_4_ aqueous solution and 1 mL of 0.4 mol L^−1^ potassium iodide (KI) aqueous solution were added to obtained solution, and kept for 30 min. The H_2_O_2_ molecules reacted with iodide anions (I^−^) under acidic conditions (H_2_O_2_ + 3I^−^ + 2H^+^ → I_3_^−^ + 2H_2_O) to produce triiodide anions (I_3_^−^) possessing a strong absorption at around 350 nm. The amount of I_3_^−^ was determined by means of UV–vis spectroscopy on the basis of the absorbance at 350 nm, from which the amount of H_2_O_2_ produced during each reaction was estimated.

The rate constants for H_2_O_2_ formation (K_f_) and decomposition (K_d_) over prepared catalysts were evaluated by assuming zero-order and first-order kinetics, respectively. Values of K_f_ and K_d_ are obtained by fitting the H_2_O_2_ evolution data to the following equation:

$$\left[ \text{H}_{\text{2}}\text{O}_{\text{2}} \right]\text{ = }\frac{\text{k}_{\text{f}}}{\text{k}_{\text{d}}}\text{\{1-exp(-}\text{k}_{\text{d}}\text{t)\}}$$

**Determination of apparent quantum yield.** The apparent quantum yield (AQY) was determined by a multi-channel photocatalytic reactor (PCX-500C Discover, Perfect Light). The wavelength of incident light is adjusted to 420, 450 and 485 nm, respectively. The AQY was calculated by the following formula:

$$\text{AQY= }\frac{\text{2 × }\text{n}_{\text{H}_{\text{2}}\text{O}_{\text{2}}}}{\text{n}_{\text{photons}}}\text{ ×}\text{ }\text{100\%}$$

where $\text{n}_{\text{H}_{\text{2}}\text{O}_{\text{2}}}$ is the amount of generated H_2_O_2_ and n_photons_ is the number of incident photons.

**Determination of solar-to-chemical conversion efficiency.** The solar-to-chemical conversion efficiency (SCC) was tested under AM1.5 global spectrum. The SCC was calculated by the following formula:

$$\text{SCC }\text{= }\frac{\text{∆G}_{\text{H}_{\text{2}}\text{O}_{\text{2}}}\text{ × }\text{n}_{\text{H}_{\text{2}}\text{O}_{\text{2}}}}{\text{t}_{\text{ir}}\text{ × }\text{S}_{\text{ir}}\text{ × }\text{I}_{\text{AM}}}\text{ × 100\%}$$

where $\text{∆G}_{\text{H}_{\text{2}}\text{O}_{\text{2}}}$ is the free energy for H_2_O_2_ formation (117kJ mol^-1^), $\text{n}_{\text{H}_{\text{2}}\text{O}_{\text{2}}}$ is the amount of generated H_2_O_2_, t_ir_ is the irradiation times, S_ir_ is the irradiation area. I_AM_ is the overall irradiation intensity.

**Photoelectrochemical measurements.** A standard three-electrode system was used to conduct the photoelectrochemical characterizations on a CHI760E electrochemical workstation, with a Pt electrode and an Ag/AgCl electrode as the counter and reference electrode, respectively. An FTO (active area of 1 cm^2^) electrode covered with samples was used as the working electrode. A 300 W Xe lamp was used as the light source. A rotating ring-disk electrode (RRDE) was used to evaluate the number of transferred electrons (n) and H_2_O_2_ selectivity in the ORR reaction. The RRDE tests were conducted in an O_2_-saturated 0.1M KOH solution with a rotating speed of 1600 rpm. The number of transferred electrons (n) is calculated according to the following formula:

$$\text{n = 4 × }\frac{\text{I}_{\text{d}}}{\text{I}_{\text{d}}\text{ + }\text{I}_{\text{r}}\text{/N}}$$

The selectivity of H_2_O_2_ is calculated by the following formula:

$$\text{H}_{\text{2}}\text{O}_{\text{2}}\text{\% = 200 × }\frac{\text{I}_{\text{r}}\text{/N}}{\text{I}_{\text{d}}\text{ + }\text{I}_{\text{r}}\text{/N}}\text{ × 100\%}$$

where I_r_ is the ring current, I_d_ is the disc current, and N is the collection efficiency (N = 0.41).

**Computational details.** In this work, all calculations were calculated by the first principles based on the Vienna Ab initio Simulation Package (VASP). The generalized gradient approximation (GGA) functional of Perdew, Burke, and Ernzerhof is used and a vacuum thickness more than 15 Å is adopted to separate it from its periodic model. The plane wave cutoff energy is set as 400 eV and the van der Waals (vdW) interactions are included by using the PBE-D2 functional. Furthermore, the convergence criterion of total energy and residual force are 10^−5^ eV and 0.01 eV/Å, respectively.

**Supplemental Figures and Tables**


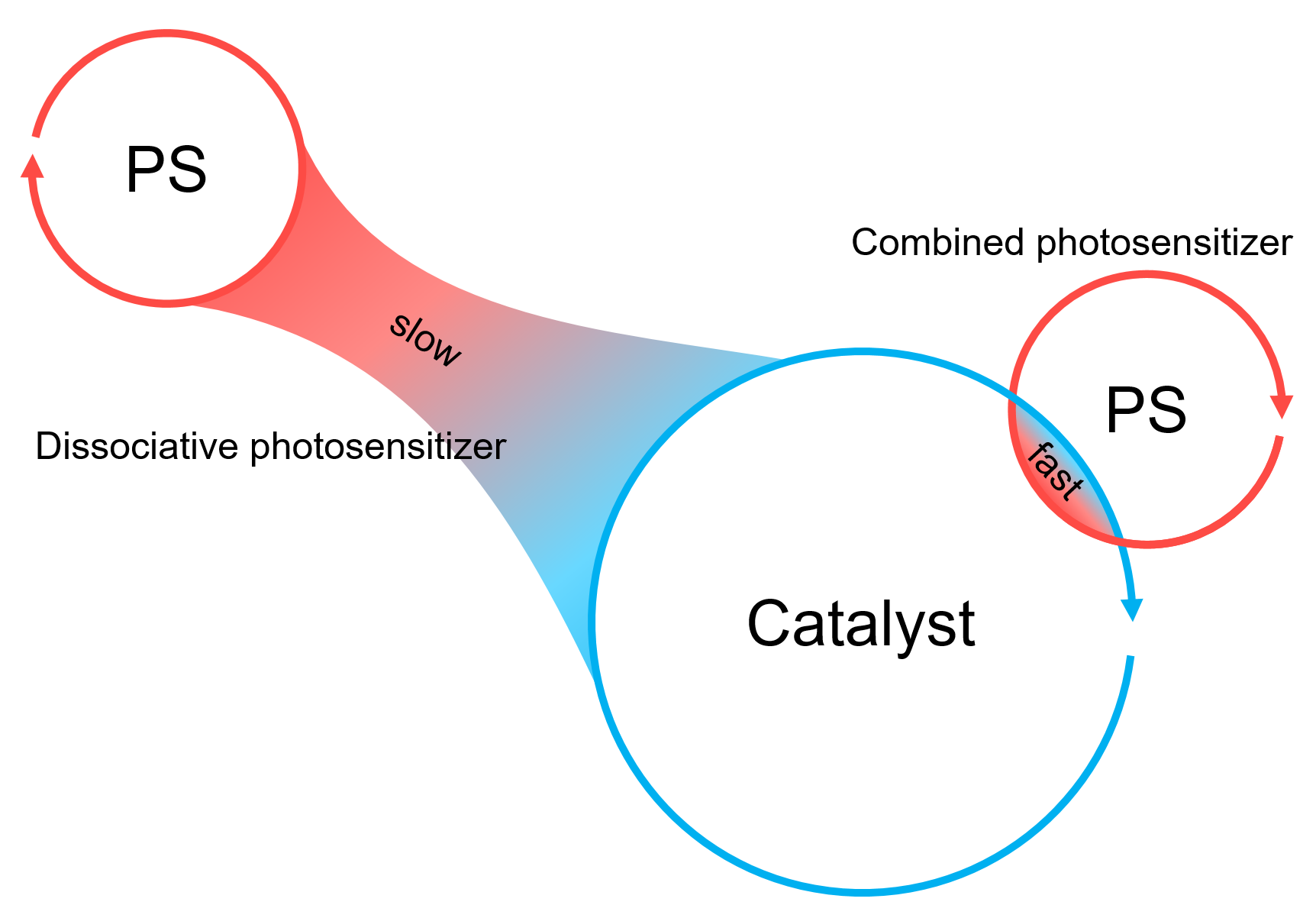


**Figure S1.** Schematic diagram of charge transfer between catalyst and photosensitizer.


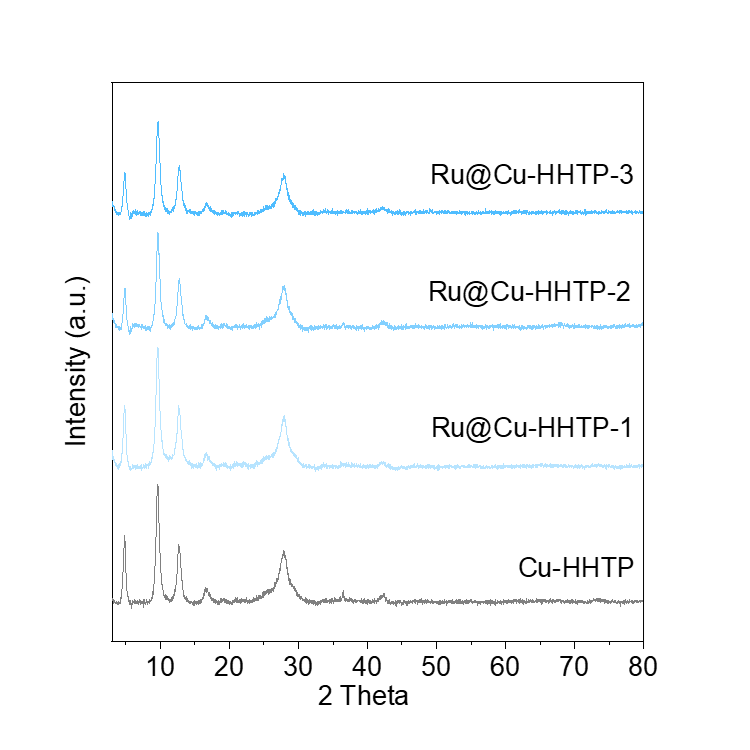


**Figure S2.** Powder XRD patterns of Cu-HHTP and Ru@Cu-HHTP samples.


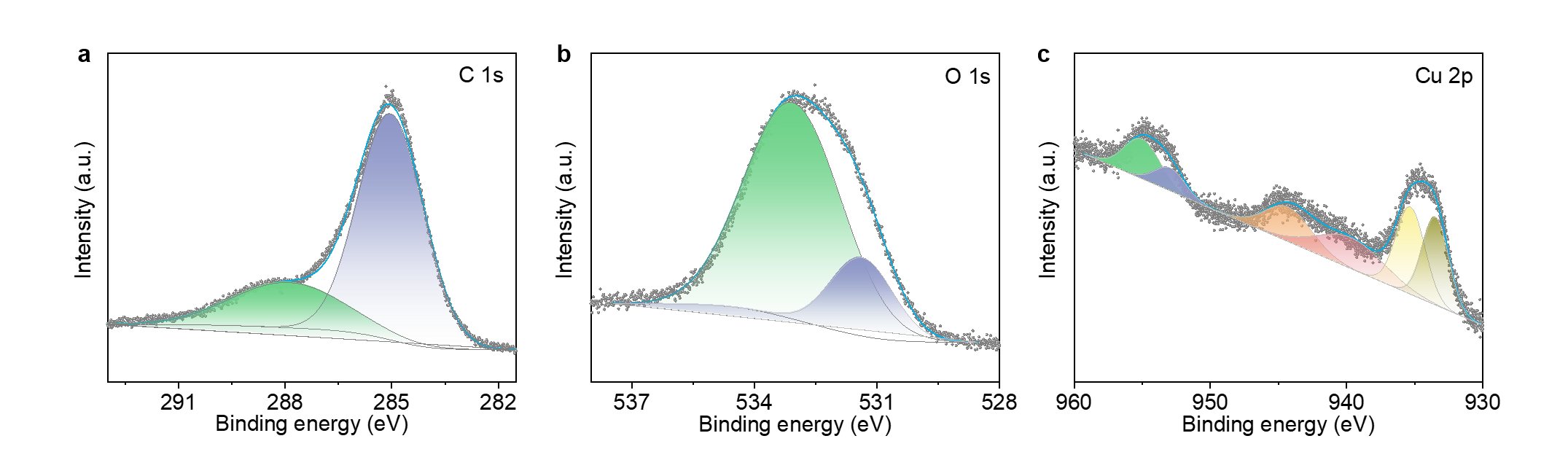


**Figure S3.** XPS spectra of Cu-HHTP. (a) C 1s XPS spectra. (b) O 1s XPS spectra. (c) Cu 2p XPS spectra.


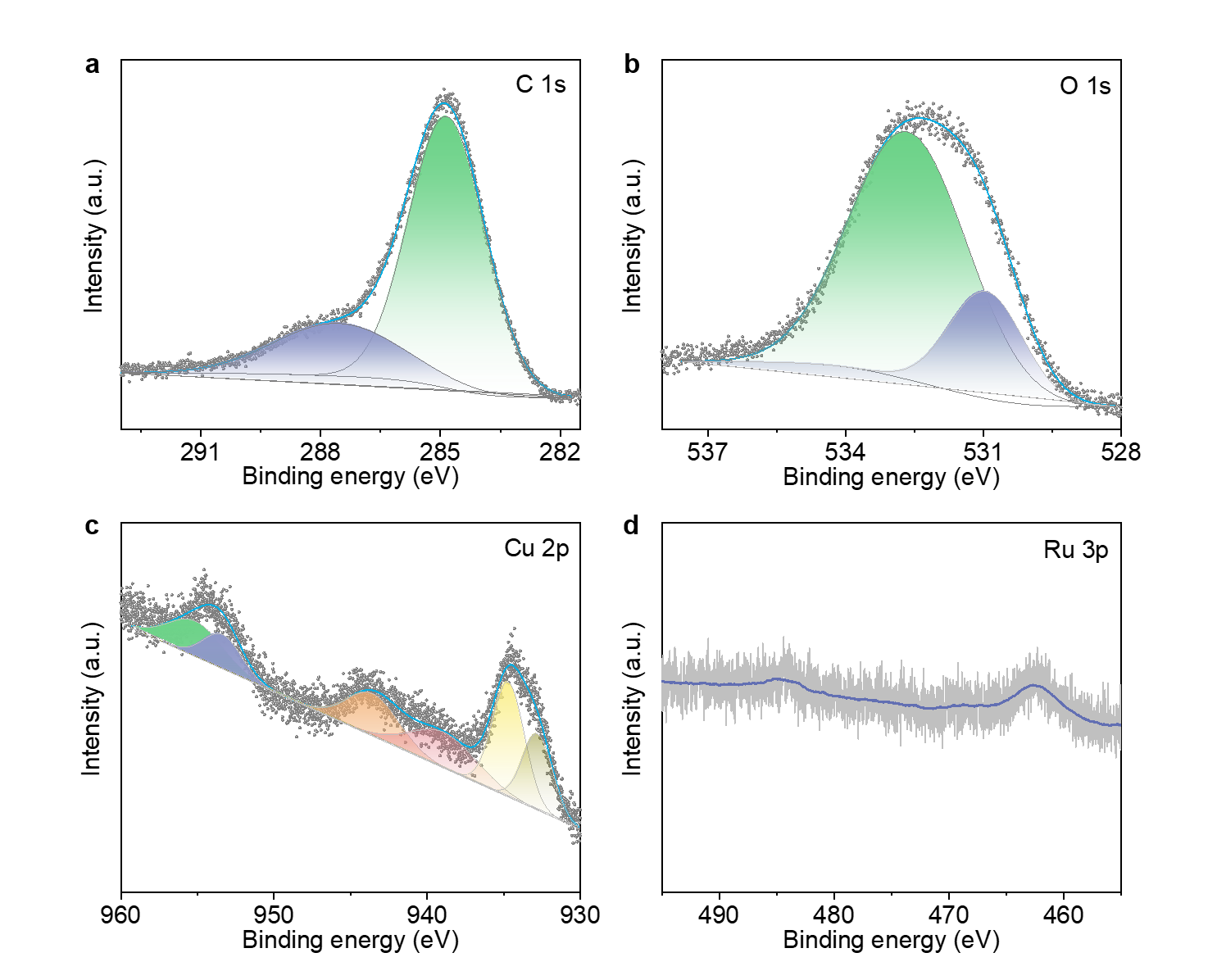


**Figure S4.** XPS spectra of Ru@Cu-HHTP. (a) C 1s XPS spectra. (b) O 1s XPS spectra. (c) Cu 2p XPS spectra. (d) Ru 3p XPS spectra.


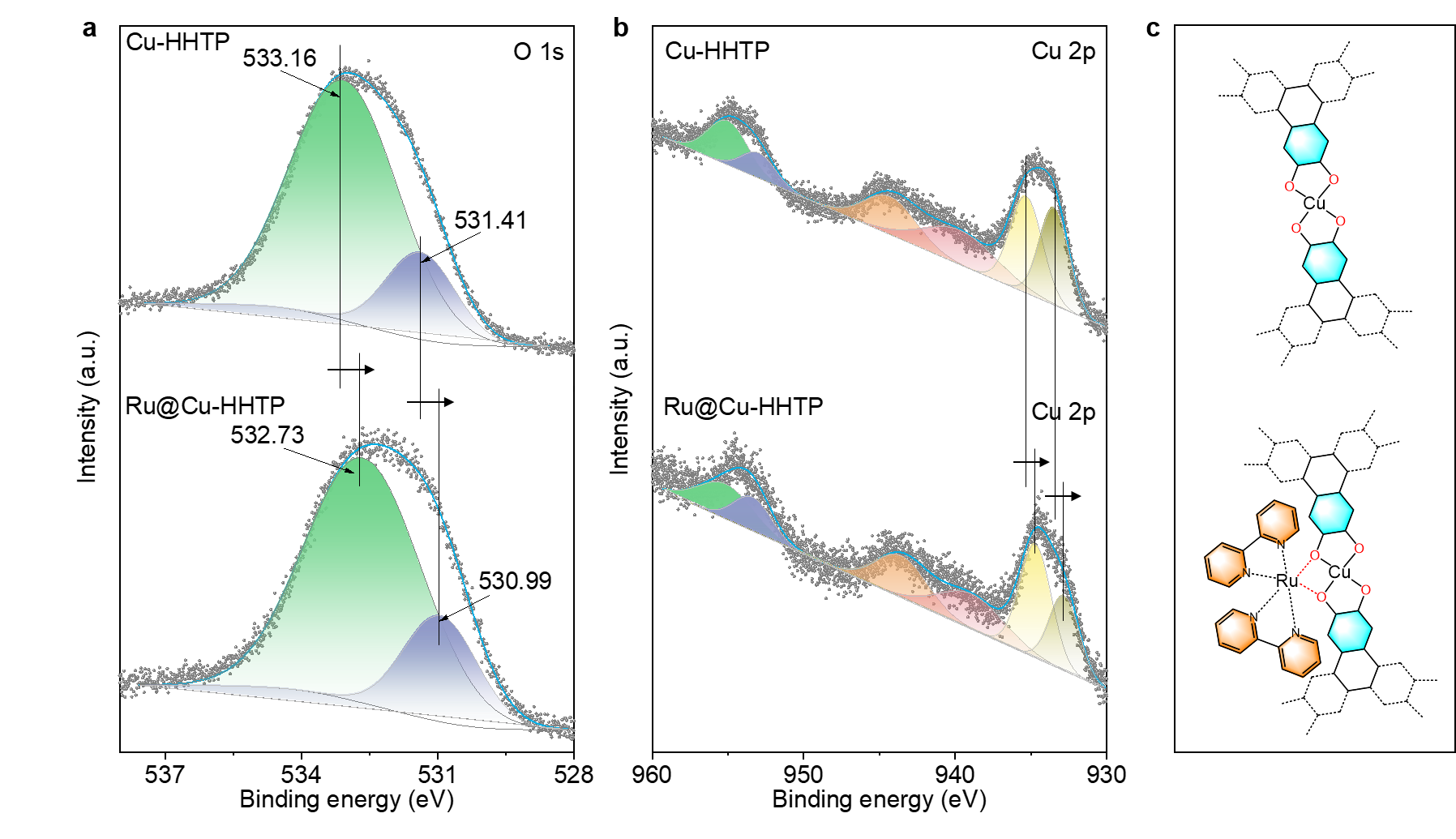


**Figure S5.** (a) Comparison of the O 1s XPS spectra of Cu-HHTP and Ru@Cu-HHTP. (b) Comparison of the Cu 2p XPS spectra of Cu-HHTP and Ru@Cu-HHTP. (c) Diagram of the local coordination structures of Cu-HHTP and Ru@Cu-HHTP.

Compared to Cu-HHTP, both the O 1s and Cu 2p peaks in Ru@Cu-HHTP shift toward lower binding energies. According to the structure of Ru@Cu-HHTP, Ru coordinates with the O in the Cu-O_4_ sites, thus O gains valence electrons from Ru, leading to an increase in its electron density. This simultaneously weakens electron-withdrawing ability of O toward Cu, resulting in a reduction in the oxidation state of Cu.


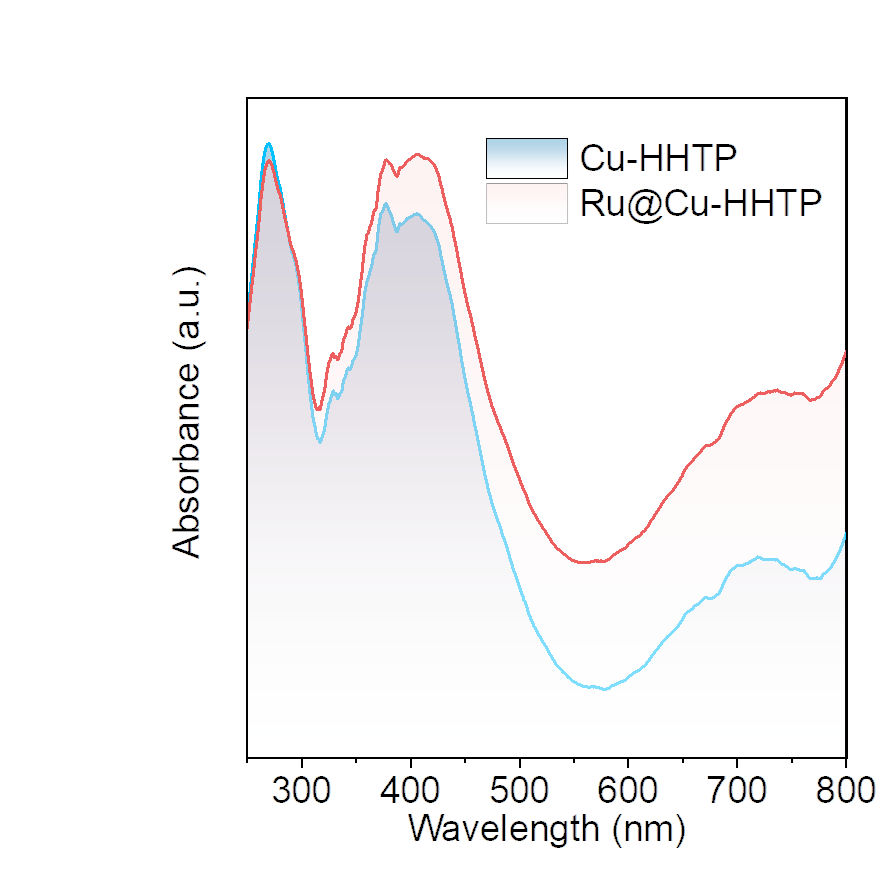


**Figure S6.** UV-vis diffuse reflectance spectra of Cu-HHTP and Ru@Cu-HHTP.


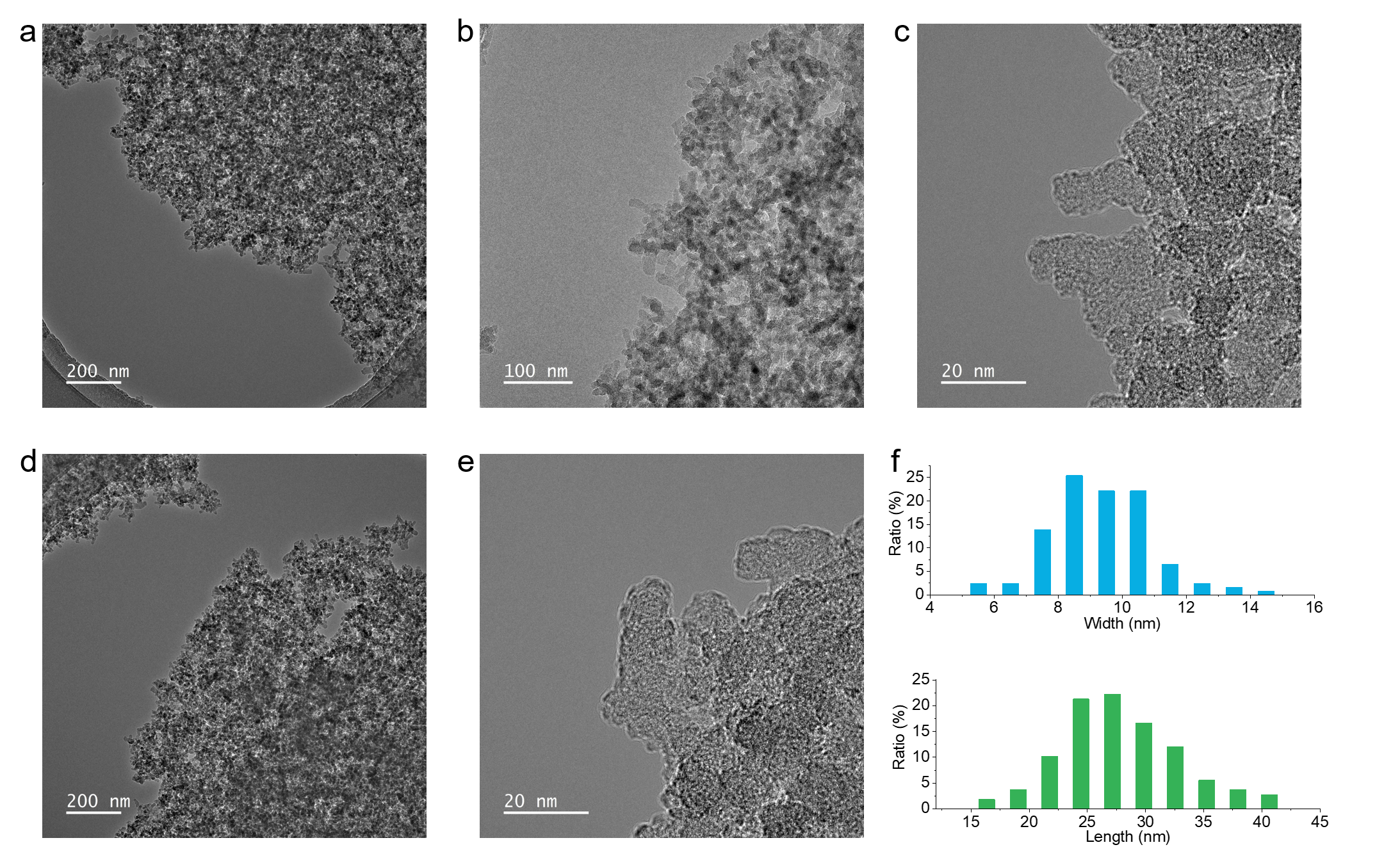


**Figure S7.** (a-c) TEM images of Cu-HHTP. (d-e) TEM images of Ru@Cu-HHTP. (f) Particle size distribution of Ru@Cu-HHTP.

Particle size statistics indicate that the sample is composed of nanocrystals with an average size of 27 nm in length and 9 nm in width, showing a uniform size distribution. The small particle size of the catalyst facilitates a larger specific surface area and reduces the difficulty of substrate molecules contacting the internal reactive sites through the MOF pores. Additionally, the uniform particle size distribution enhances the controllability of the reaction and ensures that all particles participate in the reaction with similar efficiency, thus improving the overall catalytic activity.


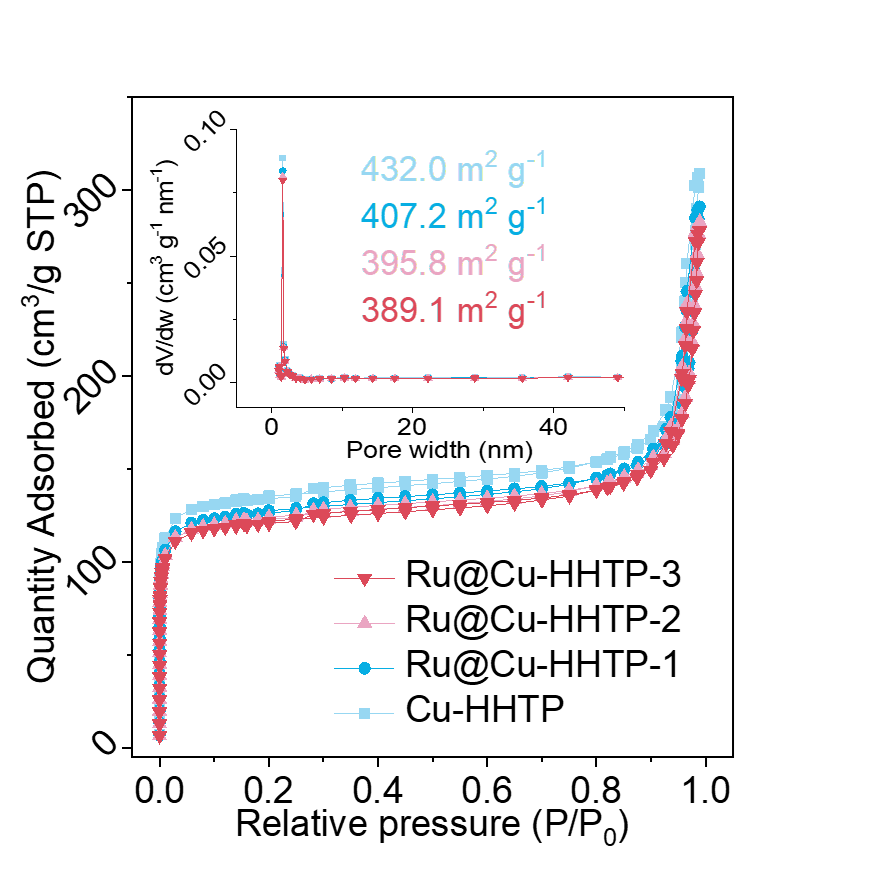


**Figure S8.** N_2_ sorption isotherms and pore size distribution of Cu-HHTP and Ru@Cu-HHTP samples.


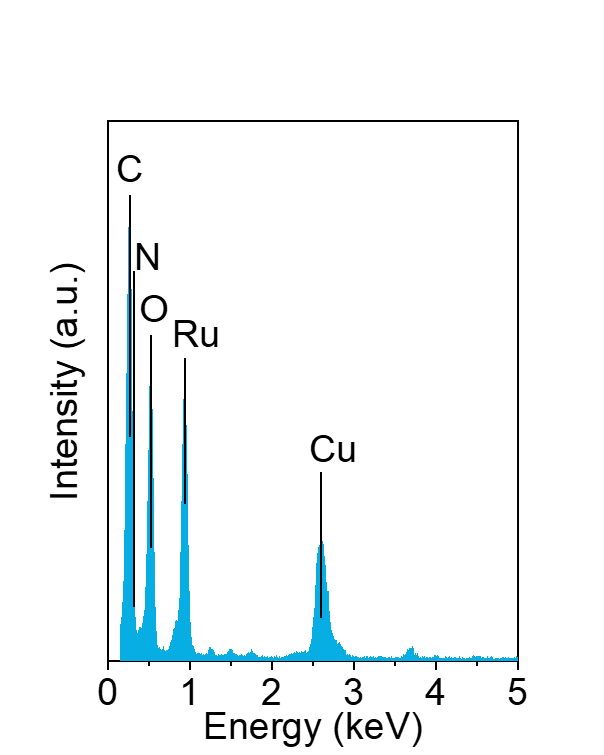


**Figure S9.** EDS analysis of Ru@Cu-HHTP.


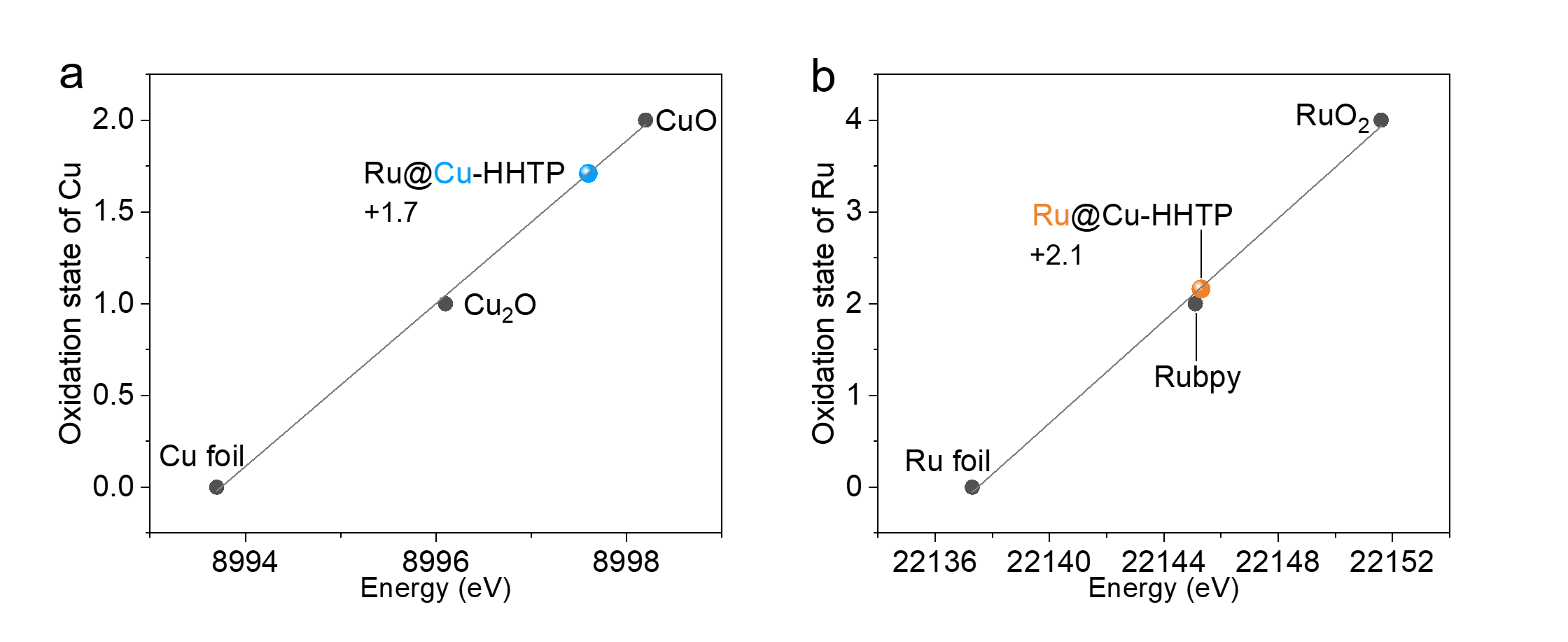


**Figure S10.** The fitted average oxidation states of (a) Cu and (b) Ru from XANES spectra


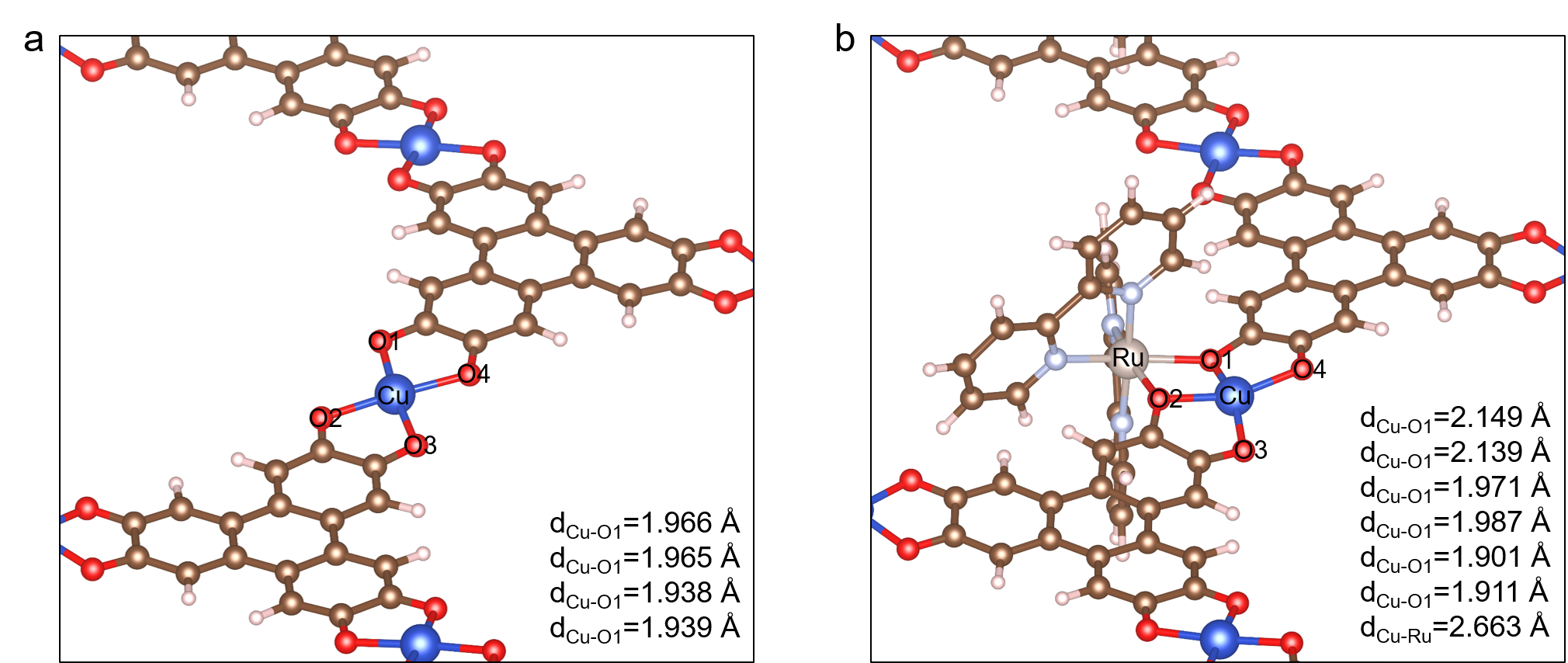


**Figure S11.** Structure optimized according to density functional theory. (a) Cu-HHTP and (b) Ru@Cu-HHTP.


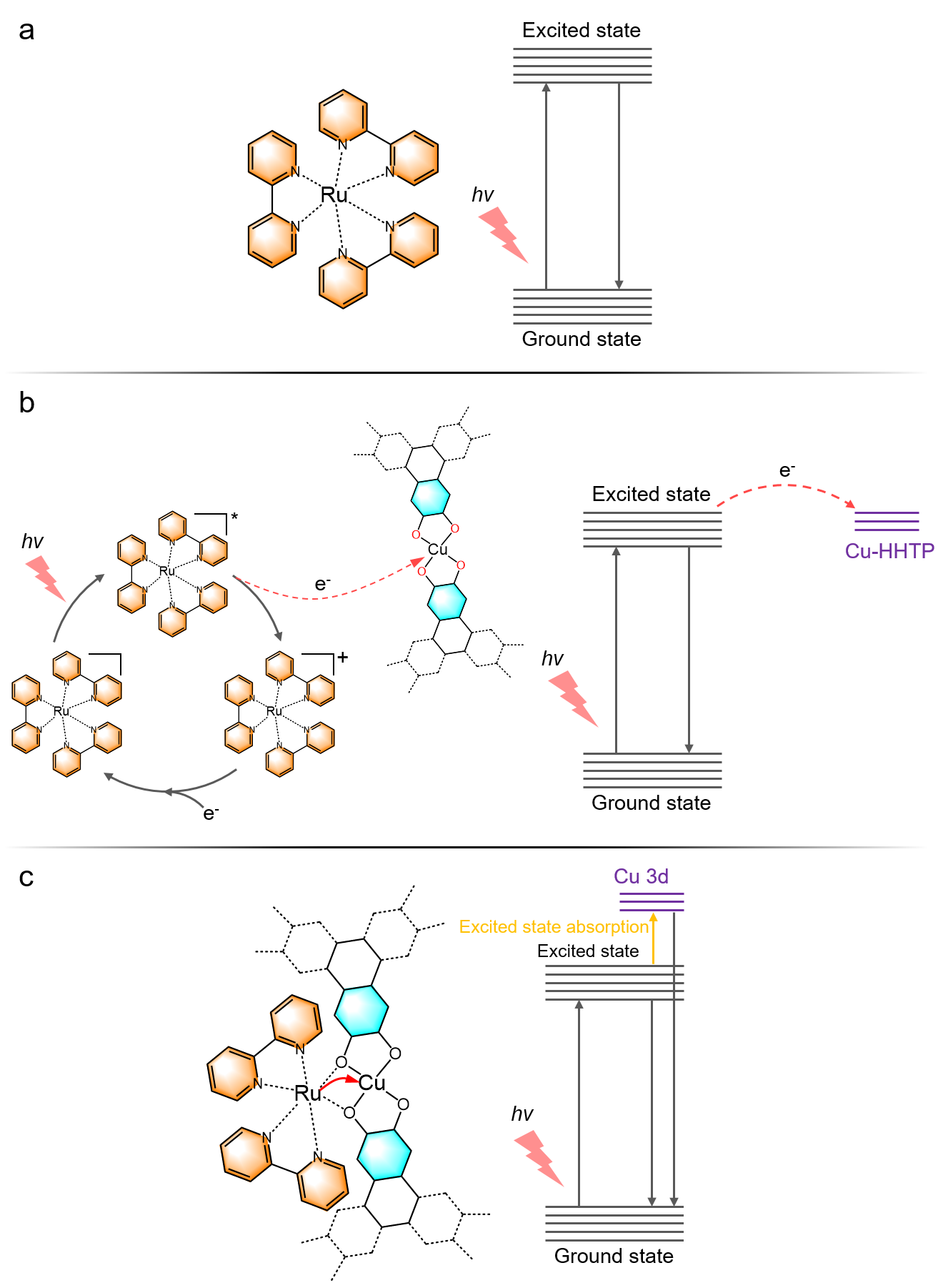


**Figure S12.** Comparison of the charge excitation process between (a) **Ru**, (b) [**Ru** + Cu-HHTP] and (c) Ru@Cu-HHTP.


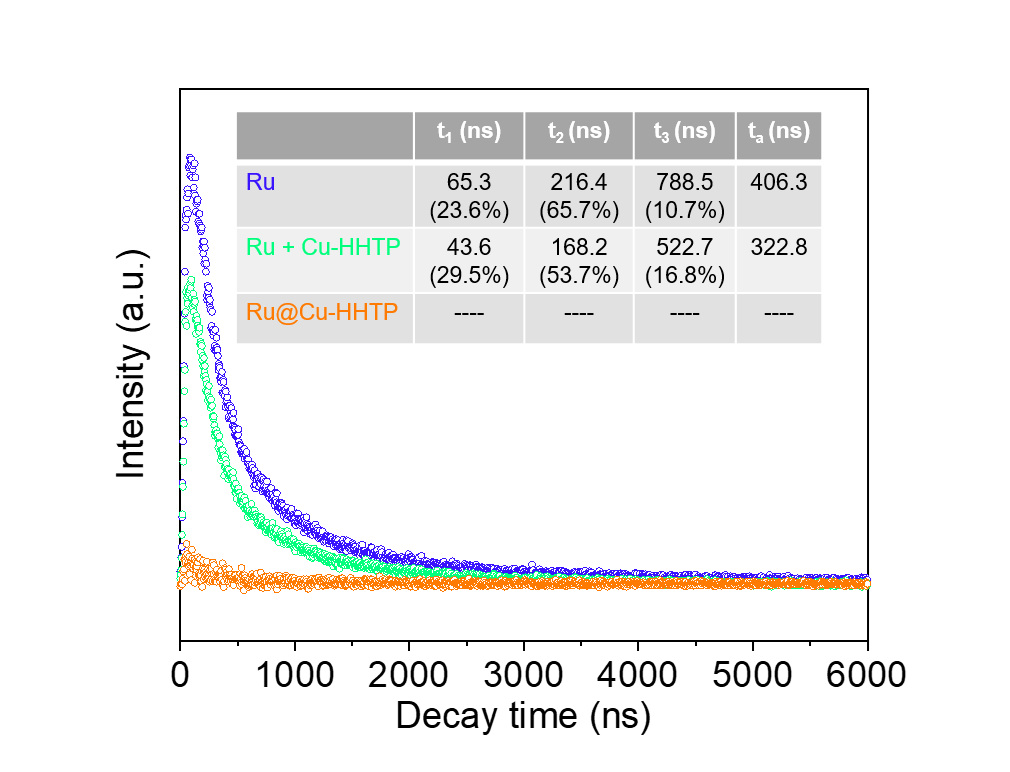


**Figure S13.** Time-resolved photoluminescence spectra of **Ru**, [**Ru** + Cu-HHTP] and Ru@Cu-HHTP.

The average emission lifetime (t_a_) was calculated by the following equation.

t_a_ = (A_1_t_1_^2^ + A_2_t_2_^2^ +A_3_t_3_^2^)/( A_1_t_1_ + A_2_t_2_ +A_3_t_3_)

The emission lifetime of Ru@Cu-HHTP is extremely short and close to the detection limit of the instrument; therefore, it is not possible to obtain a reliable fit for the lifetime value.


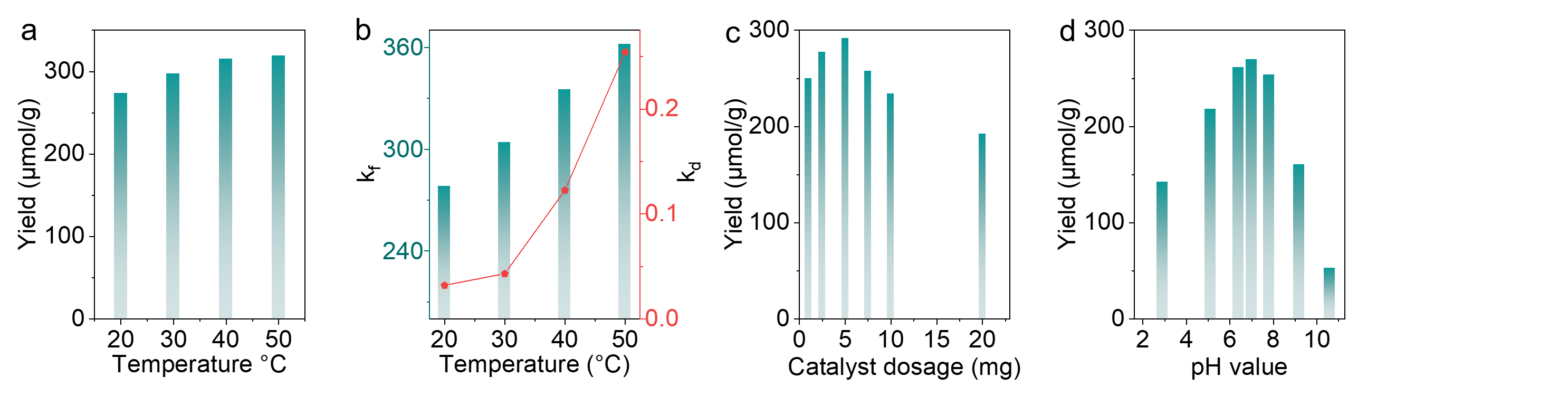


**Figure S14.** (a) Photocatalytic H_2_O_2_ production and (b) the fitted formation rate constants (*K_f_*) and decomposition rate constants (*K_d_*) over Ru@Cu-HHTP catalyst with different temperature. Photocatalytic H_2_O_2_ production with different (c) catalyst dosages and (d) pH value. Except for the investigated parameters, all other conditions were kept constant. The reaction time was 1 hour.

To investigate the effects of reaction conditions on catalytic activity, including system temperature, catalyst loading, and solution pH, we conducted a series of control experiments. Considering that the temperature increase due to xenon lamp illumination does not exceed 50°C, we used a water-circulating temperature control system to study temperatures ranging from 20 to 50°C. Kinetic fitting shows that although the increase in temperature intensifies the decomposition of H_2_O_2_, the promotion effect on the generation of H_2_O_2_ is more significant, so the net production of H_2_O_2_ increases (Figure S14b). As shown in Figure S14a, increasing the system temperature improves catalytic activity to some extent. Figure S14c indicates that the catalytic activity is optimal with a catalyst loading of 5 mg. Figure S14d demonstrates that a neutral or mildly acidic environment favors the photocatalytic reduction of O₂ to H_2_O_2_. To ensure comparability with previously reported work and based on the above performance optimization parameters, we set the experimental conditions in this study to a temperature of 20°C, a catalyst loading of 10 mg, and a solution pH of 7.


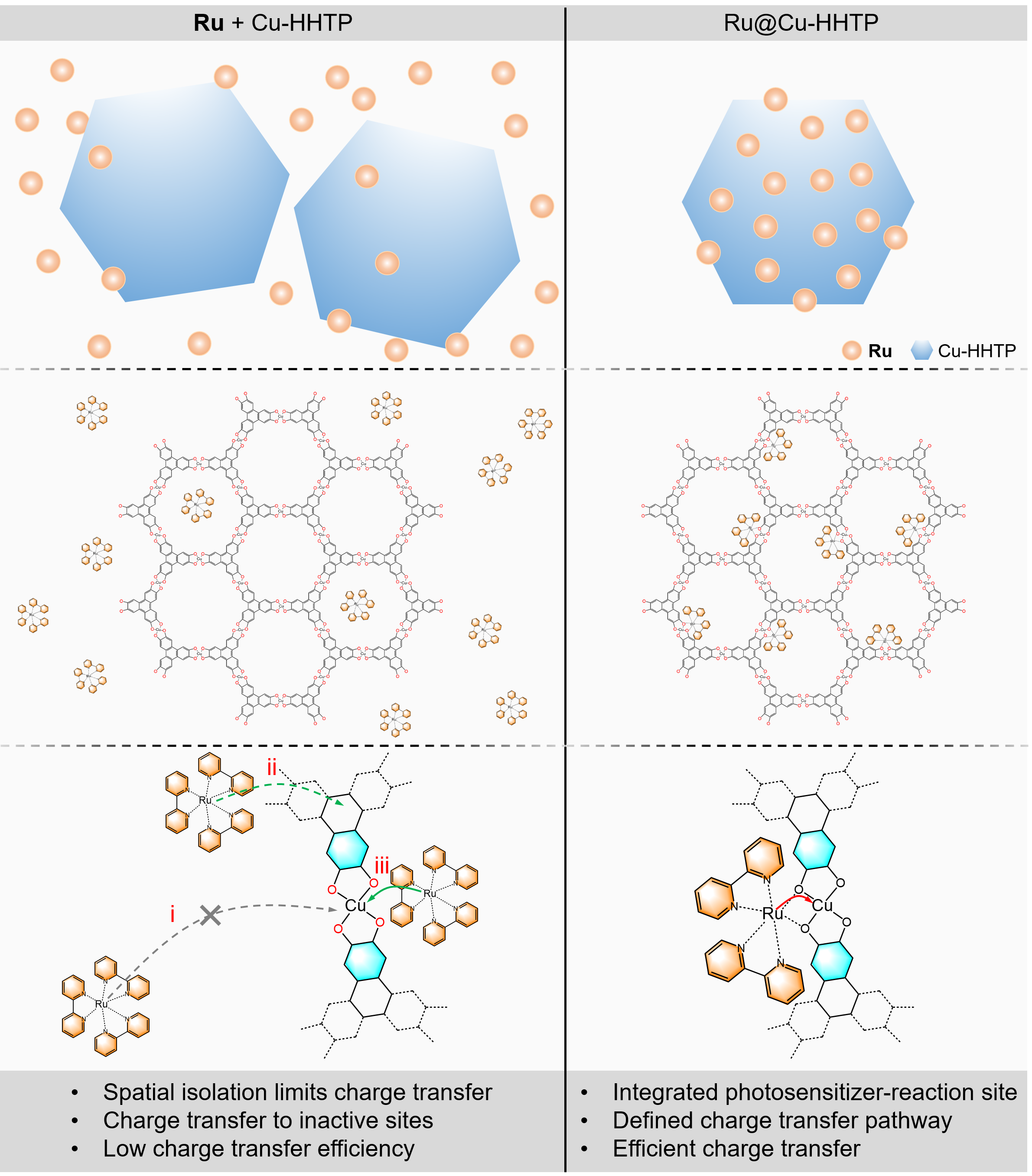


**Figure S15.** The comparison of charge transfer mechanisms between [**Ru** + Cu-HHTP] and Ru@Cu-HHTP.

In traditional photosensitizer/catalyst mixed systems, electron transfer between the photosensitizer and the catalyst relies on random contact. Since photogenerated charge carriers recombine within a short time after excitation, large quantity charge carriers on photosensitizers that do not promptly contact the catalyst cannot be effectively utilized. Furthermore, as the contact points between the photosensitizer and the catalyst are random, some charge carriers may transfer to non-active sites on the catalyst, rendering them unable to drive the reaction. As a result, the charge transfer efficiency between the photosensitizer and the catalyst in conventional mixed systems is extremely low. In previously reported similar systems, it is often necessary to add a photosensitizer in quantities several times greater than the mass of the catalyst to compensate for the inefficiency of charge transfer.

For the Ru@Cu-HHTP developed in this work, the precise alignment of the photosensitizer component with the active reaction sites enables the efficient and direct transfer of photogenerated charge carriers to the reaction sites. This design prevents charge carrier losses caused by insufficient contact or transfer to non-reactive sites, thereby enabling efficient photogenerated charge carrier transfer and utilization.


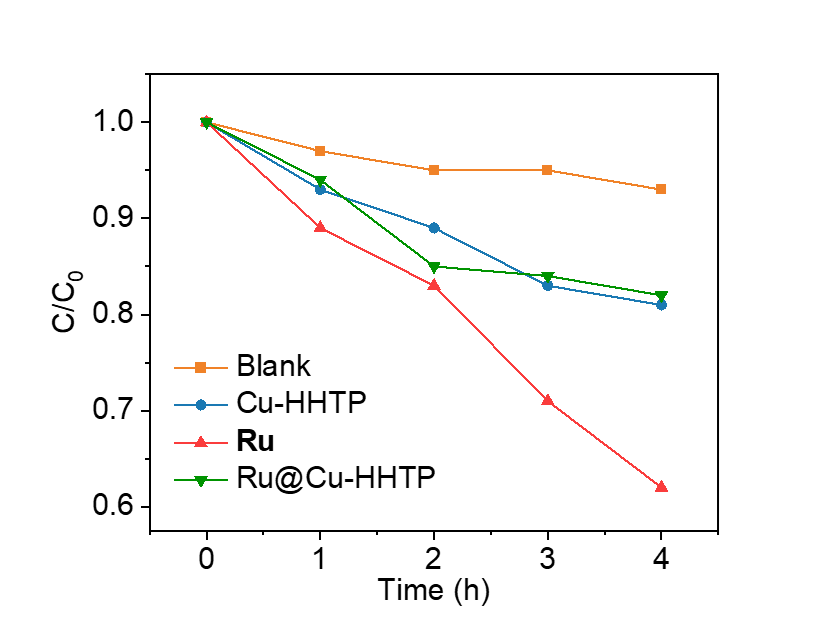


**Figure S16.** The decomposition of H_2_O_2_ in the presence different samples.


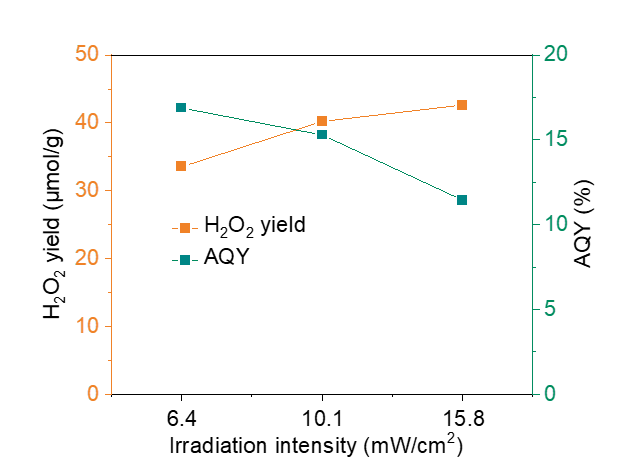


**Figure S17.** Apparent quantum yield under different irradiation intensity.


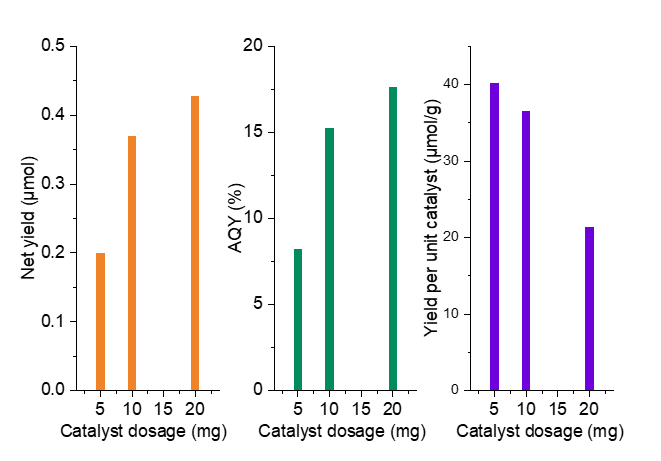


**Figure S18.** Net H_2_O_2_ yield, AQY and unit catalyst yield at different catalyst dosages.


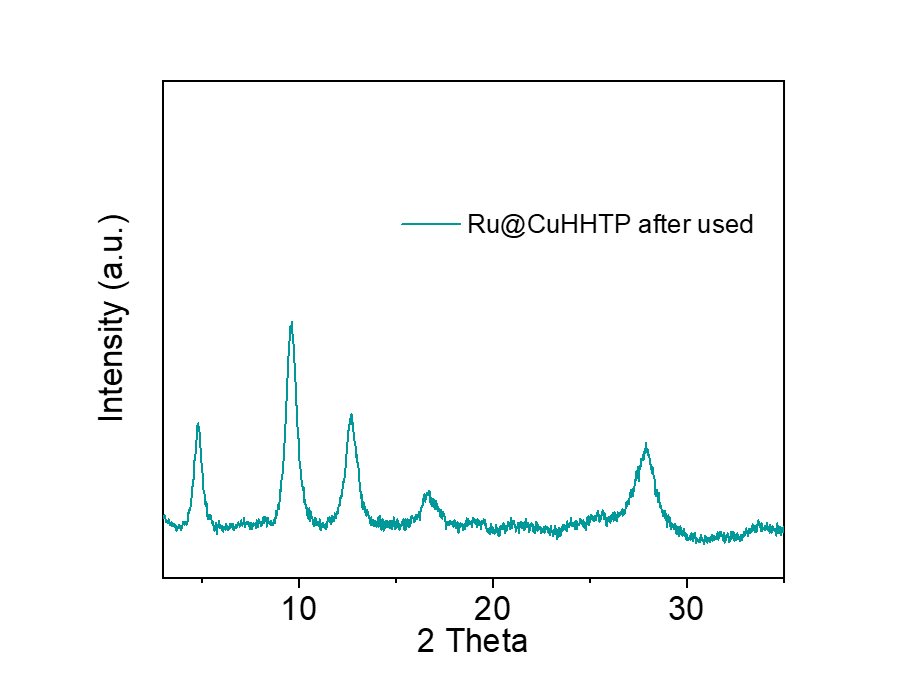


**Figure S19.** XRD pattern of Ru@Cu-HHTP sample after long-term use in the flat-plate reactor.


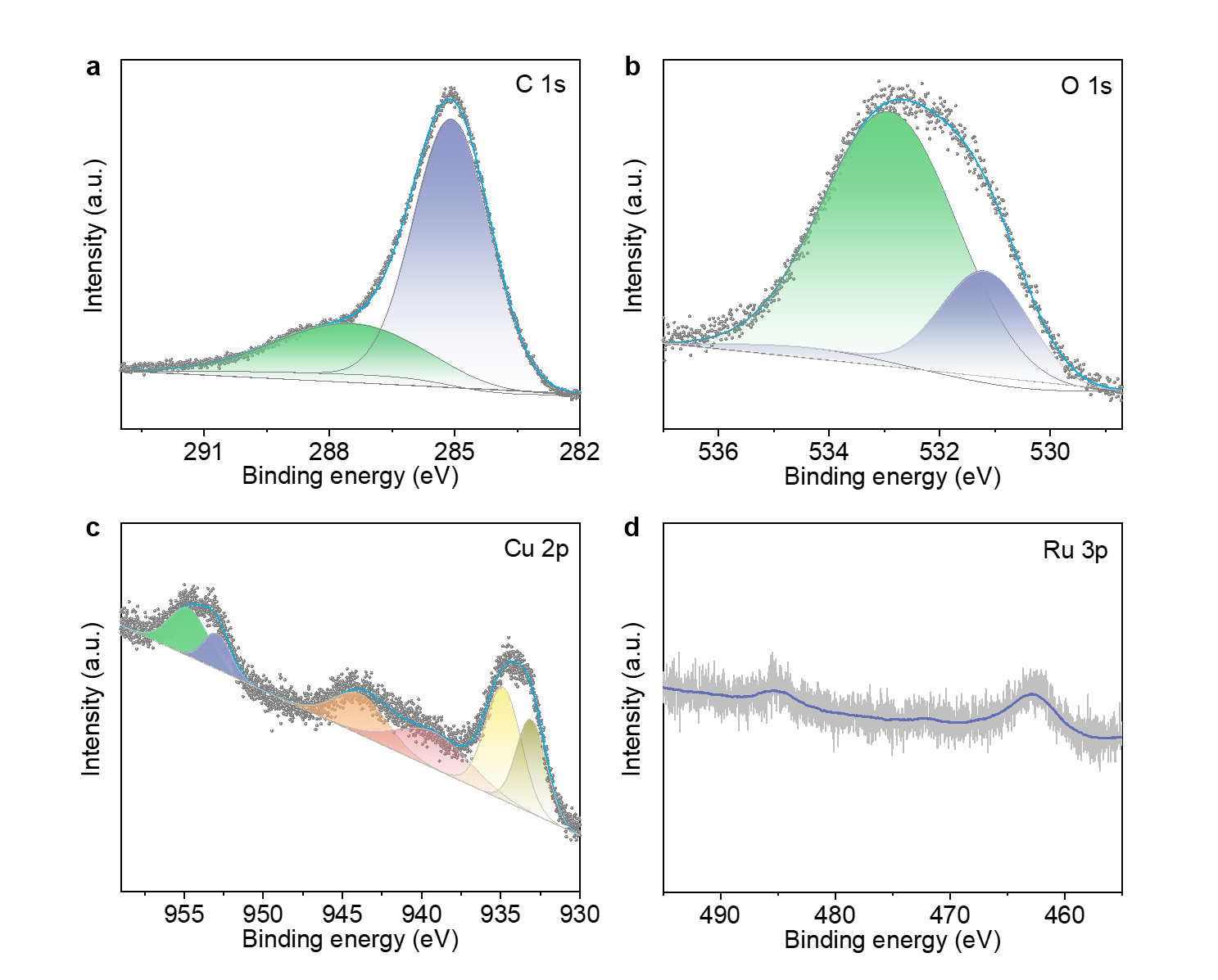


**Figure S20.** XPS spectra of Ru@Cu-HHTP sample after long-term use in the flat-plate reactor. (a) C 1s XPS spectra. (b) O 1s XPS spectra. (c) Cu 2p XPS spectra. (d) Ru 3p XPS spectra.


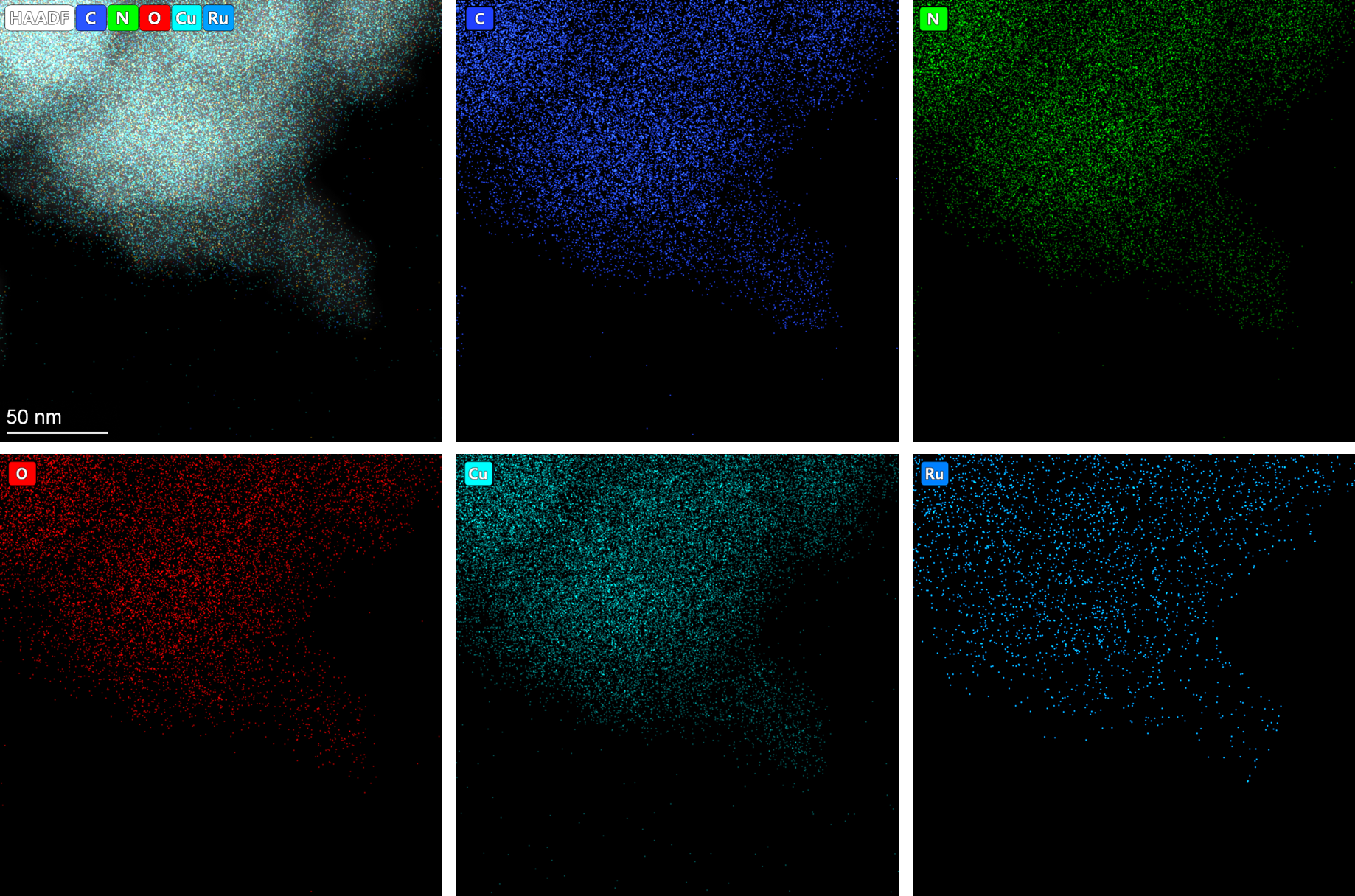


**Figure S21.** STEM-EDS elemental mapping images of Ru@Cu-HHTP after sample after long-term use in the flat-plate reactor.


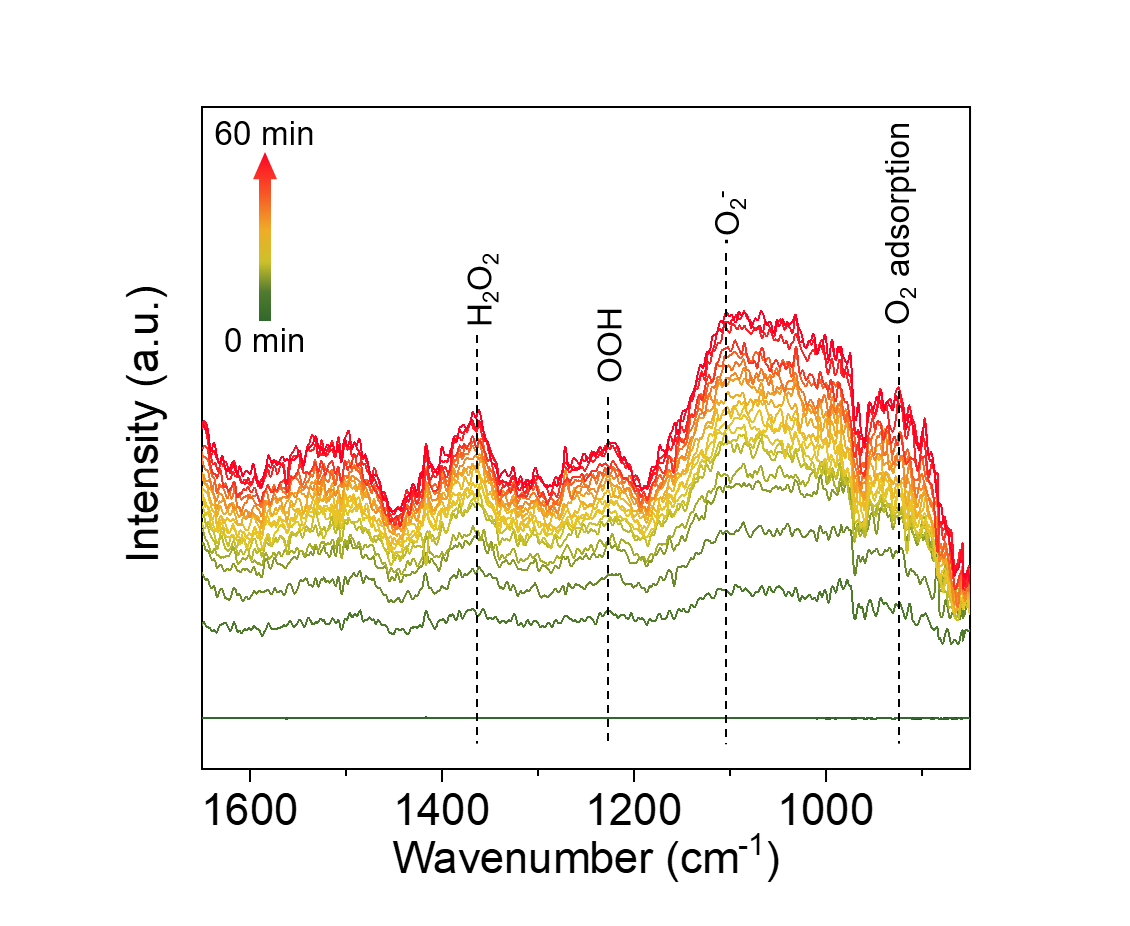


**Figure S22.** In situ DRIFTS spectra of O_2_ photocatalytic reduction over Ru@Cu-HHTP.

**Table S1.** The atomic ratio of Cu and Ru in the sample determined by ICP

| Sample | Cu:Ru (atomic ratio) |
| --- | --- |
| Ru@Cu-HHTP-1 | 1:0.05 |
| Ru@Cu-HHTP-2 | 1:0.09 |
| Ru@Cu-HHTP-3 | 1:0.16 |
| Ru@Cu-HHTP-3-after used | 1:0.14 |
